# Zoo-Sanitary Situation Assessment, an Initial Step in Country Disease Prioritization Process: Systematic Review and Meta-Analysis from 2000 to 2020 in Cameroon

**DOI:** 10.3390/pathogens12091076

**Published:** 2023-08-24

**Authors:** Mohamed Moctar Mouliom Mouiche, Eugenie Elvire Nguemou Wafo, Serge Eugene Mpouam, Frédéric Moffo, Jean Marc Kameni Feussom, Arouna Njayou Ngapagna, Youssouf Mouliom Mfopit, Claude Saegerman, Mamoudou Abdoulmoumini

**Affiliations:** 1 USAID’s Infectious Disease Detection and Surveillance (IDDS), ICF, Yaoundé P.O. Box 8211, Cameroon; mouichemoctar4@gmail.com; 2 School of Veterinary Medicine and Sciences, University of Ngaoundéré, Ngaoundéré P.O. Box 454, Cameroon; eugenieelvire@gmail.com (E.E.N.W.); sempouam@yahoo.fr (S.E.M.); freddymoffo@gmail.com (F.M.); mamoudou.abdoulmoumini@gmail.com (M.A.); 3 Epidemiology-Public Health-Veterinary Association (ESPV), Yaoundé P.O. Box 15670, Cameroon; mfeussom@gmail.com (J.M.K.F.); anjayoungapagna@gmail.com (A.N.N.); 4 IMMANA Fellow, Friedman School of Nutrition Science and Policy, Tufts University, Boston, MA 02111, USA; 5 Ministry of Livestock, Fisheries and Animal Industries (MINEPIA), Yaoundé P.O. Box 8211, Cameroon; 6 Unit of Veterinary Public Health and Clinical Sciences, Faculty of Veterinary Medicine, Universite des Montagnes, Bangangté P.O. Box 208, Cameroon; 7 Veterinary Research Laboratory, Institute of Agricultural Research for Development, Wakwa Regional Center, Ngaoundéré P.O. Box 65, Cameroon; youssoufmfopit@yahoo.fr; 8 Faculty of Veterinary Medicine, Unit of Epidemiology and Risk Analysis Applied to Veterinary Science (UREAR-ULiège), Fundamental and Applied Research for Animals & Health (FARAH) Center, University of Liege, 4000 Liege, Belgium

**Keywords:** infectious diseases, livestock, Cameroon, systematic review, meta-analysis, disease control, disease prioritization

## Abstract

To prevent and/or control infectious diseases in animal and human health, an appropriate surveillance system based on suitable up-to-date epidemiological data is required. The systematic review protocol was designed according to the PRISMA statement to look at the available data on infectious diseases of livestock in Cameroon from 2000–2020. Data were searched through online databases. Grey literature was comprised of dissertations and theses from veterinary higher education institutions in Cameroon. A random-effects model was used to calculate pooled prevalence using Comprehensive Meta-Analysis Software. Based on disease prevalence, major infectious diseases of livestock in Cameroon were gastrointestinal parasitosis (57.4% in cattle, 67.2% in poultry, 88% in pigs), hemoparasites (21.6% in small ruminants, 19.7% in cattle), bovine pasteurellosis (55.5%), fowl salmonellosis (48.2%), small ruminant plague (39.7%), foot-and-mouth disease (34.5% in cattle), and African swine fever (18.9%). Furthermore, other important endemic zoonoses in the country included: Rift Valley fever (10.9% in cattle, 3.7% in small ruminants), brucellosis (7% in cattle, 8% in pigs), bovine tuberculosis (4.7% in cattle), hepatitis E virus (8.4% in pigs) and bovine leptospirosis (2.5%). Most of the retrieved research were carried out in the Adamawa, Northwest, and West regions of Cameroon. The evaluation of existing data as evidence, albeit publication-specific, is an important step towards the process of prioritizing animal diseases, including zoonoses.

## 1. Introduction

Infectious diseases are a significant burden on animal and human health, particularly in Low- and Middle-Income Countries (LMICs) [1] where animal health public services lack resources and countries borders are porous despite important human and animal movements between neighboring countries [2].

For example, the contagious bovine pleuropneumonia (CBPP) is estimated to cost almost €45 million per year in lost productivity in twelve sub-Saharan African countries [3]. Zoonotic diseases account for 2.5 billion cases of human illness and 2.7 million human deaths worldwide each year [4]. During the last five years, two major epizootic outbreaks, African swine fever from 2014–2016 [5] and avian influenza in 2016 [6], have significantly reduced the national headcount flock size in Cameroon and contributed to financial losses worth billions of CFA francs (1$ = 550 CFA). The national objectives with regards to the livestock sector have not been achieved due to several constraints, including animal diseases, which negatively influence livestock productivity [7].

Cameroon is located in the Congo Basin, one of the five areas at high risk of disease emergence in the world [8]. Moreover, the country is exposed to numerous transboundary diseases due to the porous nature of its borders [2]. In order to prevent and control endemic and zoonotic diseases at the human/animal–environment interface, an appropriate surveillance system based on quality epidemiological data is required [9]. Furthermore, such data are valuable inputs for appropriate disease prioritization aiming at effective and efficient prevention and/or control measures at the farm, local, national, and regional level [10,11]. Meanwhile, epidemiological data in Cameroon is sparse and scarce in some regions due to difficulties of getting surveillance systems to function effectively [12]. Several studies have been conducted to determine the prevalence of specific diseases in some regions, without providing national estimates of these diseases. Recent systematic review studies carried out by Asante et al. [13] on important zoonotic bacteria in Africa did not include any information from Cameroon and this was justified by the scarcity of published studies online at the national level. In the absence of a global view of the health situation and surveillance capacities of the country, it may be difficult to identify priority diseases and characterize drivers of emerging and re-emerging diseases. Likewise, a lack of data on disease burden may lead to underestimation of disease impact or effect with important consequences on the implementation of Cameroon’s Animal Health and Veterinary Public Health Policy. It is in this light that the present study was initiated, to systematically review and further analyze available data retrieved online from published peer-reviewed articles and valuable theses on infectious diseases in livestock in Cameroon as an epidemiological contribution for effective interventions and prevention/control strategies.

## 2. Results

### 2.1. General Characteristics and Distribution of Studies Included in the Review

**Figure 1:**
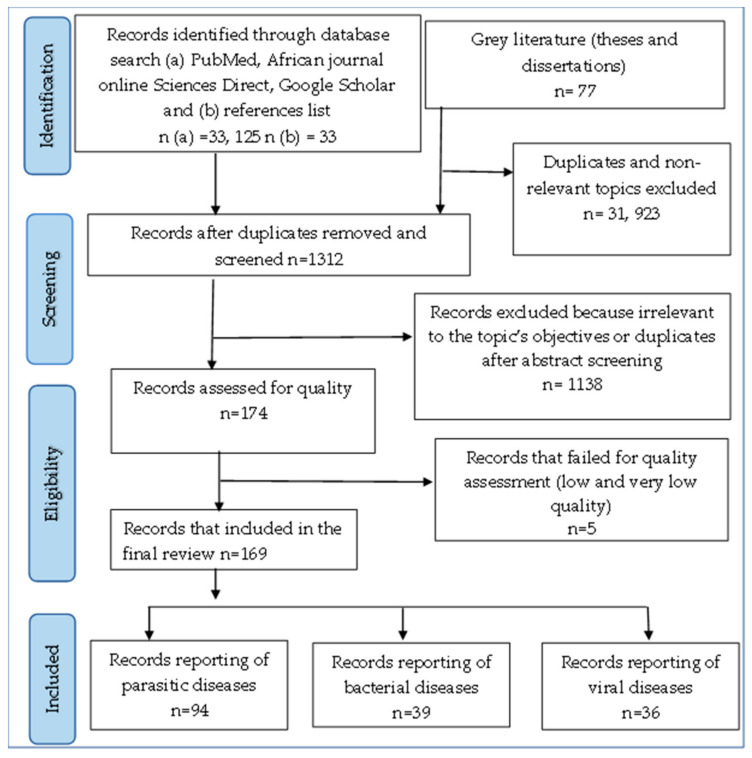
PRISMA flow-chart illustrating the study selection process for infectious diseases of production animals in Cameroon.

**Figure 2:**
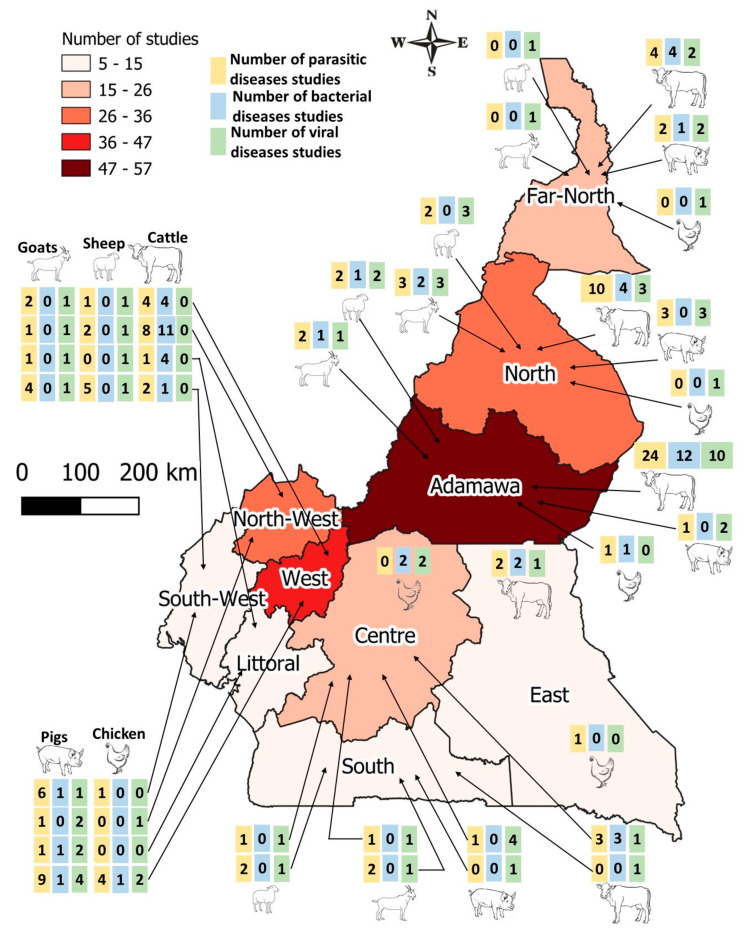
Map of Cameroon showing geographical distribution of number of disease studies (parasitic, bacterial, viral) with respect to animal species (cattle, sheep, goats, pigs, chicken).

From a total of 33,235 citations identified during preliminary research, and after different screening stages (Figure 1), 169 were rated as good quality and thus included in the final analysis. Of all 169 studies included in the review, 94 reported on the outcome of parasitic diseases, 39 reported on the outcome of bacterial diseases, and 36 on viral diseases in food-producing animals. The Adamawa region had the largest number of studies (34.54%), followed by the West region (20.6%) and the North (18.78%) region. Studies on cattle were undertaken mostly in the Adamawa (38.65%), North (15.96%), and Northwest (15.13%) regions. Studies on pigs were mostly carried out in the West (32.26%) and Southwest (16.13%) regions. The North and Southwest regions had the highest number of studies on sheep (22.22% and 18.52%, respectively) and goats (27.58% and 17.24%, respectively) while the West region hosted the largest number of studies on chicken (35%) followed by the Centre region (25%) (Figure 2). As concerns the types of samples, blood was most commonly tested (48.48%) followed by fecal swabs for parasitic diseases and live animals or carcasses for bacterial and viral diseases diagnosis. Microscopy was the most frequently used diagnostic method for parasitic infections (56.28%) while serology was used for bacterial (41.02%) and viral infections (44.44%) (Table 1).

### 2.2. Parasitic Diseases Situation of Livestock in Cameroon

Gastrointestinal parasites (58.51%) were the most frequently reported followed by hemoparasites (36.17%), ectoparasites (11.70%), and muscular/cerebral coenuruses (1.06%) (Table 2).

#### 2.2.1. Trypanosomiasis

**Figure 3:**
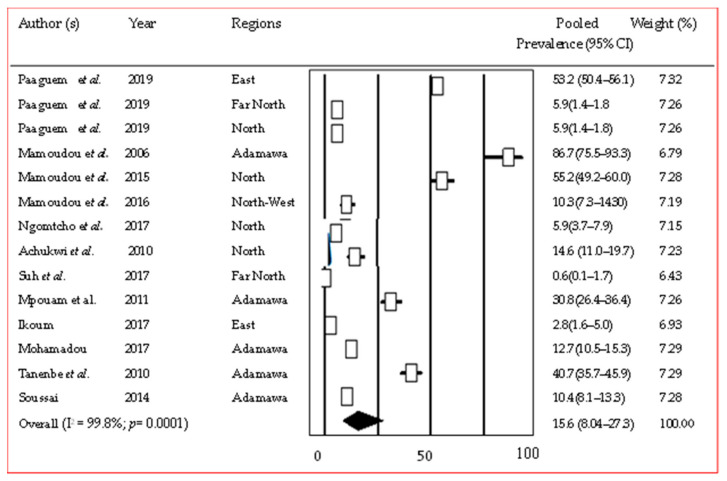
Forest plot of pooled prevalence of trypanosomiasis in cattle in Cameroon [14,15,16,17,18,19,20,21,22,23,24,25].

Trypanosomiasis infections were reported in 24 studies conducted in the Adamawa, North, and Southwest regions. *Trypanosoma congolense*, *T. brucei*, and *T. vivax* were the most frequently reported species. *T. theileri*, *T. grayi*, and *T. simaie* were reported less frequently, and only in studies where PCR was used for diagnosis (Table S1). The pooled prevalence was significantly (*p* = 0.007) higher in pigs: 54.2% [95% CI (39.6–68.2%)] (Figure S1), compared to cattle (15.6% [95% CI (8.4–27.3%)]) (Figure 3) [14,15,16,17,18,19,20,21,22,23,24,25] and small ruminants (28.4% [95% CI (12.7–51.9%)]) (Figure S2). Regional subgroup analysis for cattle gave a prevalence of 26.1% in the Adamawa region and 16.8% in the North (Table S2).

#### 2.2.2. Tick-Borne and Other Blood Parasites

Nine studies reported other blood parasites, including tick-borne parasites. *Anaplasma*, *Babesia*, and *Theileria* species were the most frequently reported (Table S3). The pooled prevalence of tick-borne parasites of 21.6% [95% CI (8.1–46.3%)] and 19.7% [95% CI (4.7–85.5%)] was obtained for small ruminants and cattle, respectively. For specific tick-borne diseases, pooled prevalence ranged between 3.5–10.9% in small ruminants and 21.6–35.9% in cattle (Table S4).

#### 2.2.3. Gastrointestinal Parasites

Gastrointestinal parasites were investigated in a total of 22 studies (Table S5). Overall, the pooled prevalence obtained in cattle (57.4% [95% CI (45.3–68.7%)]) (Figure S3) was significantly (*p* = 0.002) lower than that obtained in poultry (67.2% [95% CI (48.3–81.8)]) and pigs (88.0% [95% CI (78.2–93.8%)].

#### 2.2.4. Fascioliasis and Other Trematodes

A total of 17 studies reported findings on fascioliasis in Cameroon, a majority of which were carried out in the Adamawa (10/17). The two known species *Fasciola hepatica* and *Fasciola gigantica* were found to be present in the country (Table S6). Fascioliasis prevalence ranged between 0.6% for *Fasciola hepatica* and 66.9% for *Fasciola gigantica* with a pooled prevalence of 17.3% [95% CI (11.0–26.2%)] (Figure S4). Based on three studies, a pooled prevalence of 8.8% [95% CI (2.1–30.2%)] was obtained in small ruminants. Regional subgroup prevalence showed a significant difference (*p* = 0.006) between the Adamawa region that had the highest prevalence (41.5%), the West region (22.6%), and the Northwest region (2.4%) (Table S7). Other trematodes parasites such as *Paramphistomum* spp., *Echinostosoma* spp., *Schistosoma bovis*, and *Dicrocoelium hopes* were also reported in food-producing animals. *Paramphistomum* spp. was the most reported (Table S8) with a pooled prevalence of 12.6% [95% CI (4.5–30.8%)] in cattle, 19.7% [95% CI (3.2–64.7%)] in goats, and 23.0% [95% CI (5.4–61.0%)] in pigs (Table 2).

#### 2.2.5. Cysticercosis and Other Cestode Diseases

Cysticercosis was most reported in pigs in the West and North regions of Cameroon (Table S9). Meta-analysis using results from 10 studies gave a pooled prevalence of 6.2% [95% CI (3.3–11.3%)] for porcine cysticercosis (Figure S5). Regional subgroup analysis showed a prevalence of 21.5% [95% CI (9.9–40.6%)] in the North region, 3.2% [95% CI (1.0–9.6%)] in the West region, and 8.75% [95% CI (6.3–12.6%)] in the Centre region (Table S10). Other cestode diseases reported included monieziosis in ruminants (7 studies), *Choanotenia* spp., *Amoebotenia* spp., *Hymenolepis* spp., and *Railiettina* spp. in poultry, and coenuruses in ruminants (Table S11). A pooled prevalence of 3.6% [95% CI (2.4–5.4%)] for monieziosis in cattle was observed.

#### 2.2.6. Nematodiasis

Roundworm infestations were reported in 25 studies (Table S12). Most studies reported the infestations in cattle (11/25) and pigs (7/25). *Strongyloides* spp. (20/25), *Strongylus* spp. (14/25), and *Trichuris* spp. (13/20) were the most frequently reported species.

#### 2.2.7. Coccidiosis and Other Protozoan Parasitoses

Coccidial infections were reported in 18 studies, most of which were carried out on chicken (8/18) and pigs (5/18) and in the West region (10/18) (Table S13). Pooled prevalences of 30.2% [95% CI (10.3–61.9%)], 49.2% [95% CI (19.4–72.6%)], 51.1% [95% CI (2.8–97.5)], and 4.5% [95% CI (1.1–17.0%)] were obtained in pigs, poultry, goats, and cattle, respectively (Table 2). *Cryptosporidium* spp. was reported in cattle in the West while the only acanthocephalan parasite encountered was *Macracanthorhynchus hirudinaceus* in pigs in the West region. In all cases, the diagnosis was based on Coprological techniques.

#### 2.2.8. Ectoparasites

Ectoparasites of livestock were reported in 11 studies carried out in various regions of the country. *Ambloyamma* spp. and *Rhipicephalus* (*Boophilus*) spp. were the most frequently reported hard ticks (Table S14). From three studies that reported the tick load, a total of 27,299 ticks were collected from 675 cattle (average: 40 ticks/head). Sarcoptic scabies (mange) were also reported in pigs in the West region.

### 2.3. Bacterial Diseases

**Figure 4:**
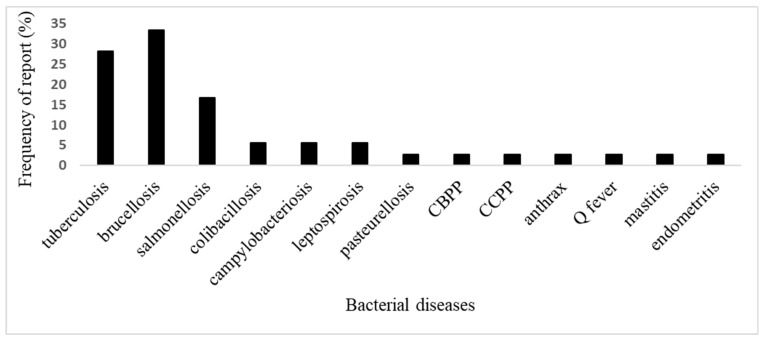
Frequency of report of various bacterial disease. Legend: CBPP: Contagious bovine Pleuropneumonia; CCPP: Contagious Caprine Pleuropneumonia.

Overall, brucellosis (33.3%), tuberculosis (28.2%), and salmonellosis (16.7%) were the most frequently reported bacterial diseases, while colibacillosis (5.6%), campylobacteriosis (5.6%), leptospirosis (5.6%), pasteurellosis (2.8%), contagious bovine pleuropneumonia (2.8%), contagious caprine pleuropneumonia (2.8%), anthrax (2.8%), Q fever (2.8%), mastitis (2.8%), and endometritis (2.8%) were less frequently reported over the whole country (Figure 4)).

#### 2.3.1. Brucellosis

Brucellosis was reported in a total of 12 studies, 10 in cattle, 1 in pigs, and another 1 in small ruminants. *Brucella* spp. was referenced in most studies, while *Brucella abortus* was reported in three studies and *Brucella melitensis* in one study. Most reports came from the Adamawa (6) and the Northwest (6) regions (Table S15). Pooled prevalences of 8.0% [95% CI (0.0–17.40%)] and 7.0% [95% CI (4.8–10.0%)] were obtained in pigs and cattle, respectively (Figure S6). Regional subgroup analysis showed a prevalence of 8.4% [95% CI (3.6–18.4%)] in the Adamawa region and 5.8% [95% CI (4.0–8.5%)] in the Northwest region (Table S16).

#### 2.3.2. Bovine Tuberculosis

**Figure 5:**
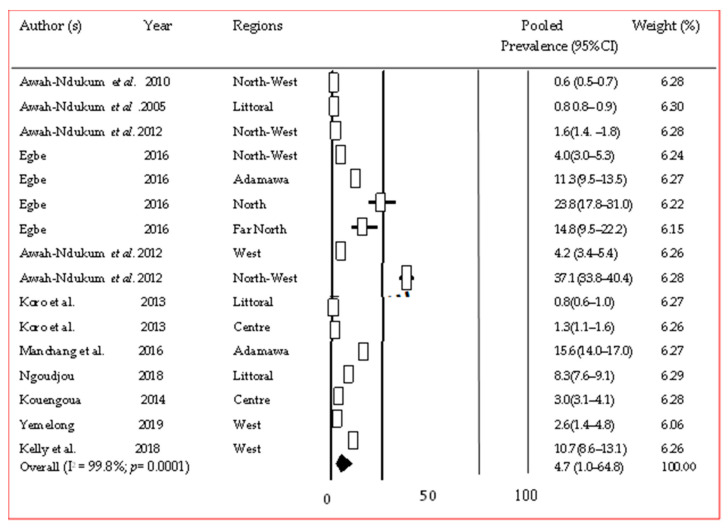
Forest plot of pooled prevalence of bovine tuberculosis in Cameroon [26,27,28,29,30,31,32,33,34,35].

A total of 11 studies described tuberculosis in animals in Cameroon (10 in cattle and 1 in goats). Various *Mycobacterium bovis* strains identified were spoligotypes SB0953, SB2162, SB2663, and SB2664 (Table S17). A pooled prevalence of 4.7% [95% CI (1.0–64.8%)] from all 12 studies was obtained in cattle (Figure 5) [26,27,28,29,30,31,32,33,34,35]. Subgroup analysis gave pooled prevalences ranging from 1.7% [95% CI (0.4–7.1%)] in the Centre region to 3.6% [95% CI (2.3–5.5%)] in the West region (Table S18).

#### 2.3.3. Salmonellosis, Colibacillosis, and Campylobacteriosis

Salmonellosis, colibacillosis, and campylobacteriosis were mainly reported in chicken, but were also reported in cattle and pigs in various regions of Cameroon (Table S19). In all cases, the diagnosis was done by bacterial culture from fecal samples. A pooled prevalence of 48.2% [95% CI (12.9–85.5%)] was obtained from three studies for salmonellosis in poultry (Table 3).

#### 2.3.4. Other Bacterial Diseases

Other bacterial infections encountered included leptospirosis, pasteurellosis, Contagious Caprine Pleuropneumonia (CCPP), Contagious Bovine Pleuropneumonia (CBPP), anthrax, Q fever, mastitis, and endometritis of infectious origin (Table S20). Combining two studies for leptospirosis and pasteurellosis in cattle gave pooled prevalences of 2.5% [95% CI (0.0–64.2%)] and 55.5% [95% CI (39.2–70.7%)], respectively (Table 3).

### 2.4. Viral Diseases

Overall, Foot-and-mouth disease (33.33%), African Swine Fever (11.11%), *Peste des Petits Ruminants* (11.11%), Rift Valley fever (8.31%), hepatitis E (5.56%), avian influenza H5N1 (5.56%), Bovine viral diarrhea (2.78%), Infectious Bovine Rhinotracheitis (2.78%), Lumpy Skin Disease (2.78%), Influenza H1N1 (2.78%), Newcastle’s Disease (2.78%), porcine hokovirus (2.78%), porcine bocavirus (2.78%), and chicken anemia (2.78%) were, in decreasing order of magnitude, the most frequently reported viral diseases (Table 4).

#### 2.4.1. Foot-and-Mouth Disease (FMD)

**Figure 6:**
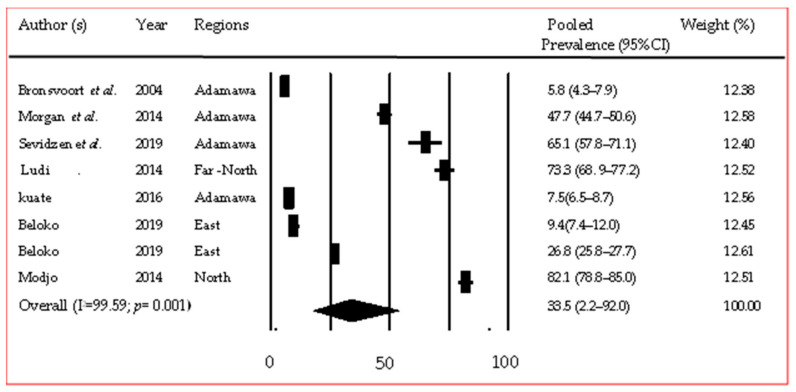
Forest plot of pooled prevalence of FMD in cattle in Cameroon [36,37,38,39,40,41,42].

Foot-and-mouth disease was reported in 13 studies and described mostly in cattle (9/13), as well as in small ruminants (3/13) and pigs (2/13). The Adamawa region hosted more studies than any other region (6/13). Five serotypes of the virus were: A, O, SAT1, SAT2, and SAT3 (Table S21). Overall, low pooled prevalence was significantly (*p* = 0.001) observed with pigs: 4.4% [95% CI (3.1–6.3%)] and sheep: 8.1% [95% CI (3.6–17.4%)] compared to cattle: 33.5% [95% CI (2.2–92.0%)] (Figure 6) [36,37,38,39,40,41,42]. Subgroup analysis for the Adamawa region gave a pooled regional prevalence of 34.8% [95% CI (8.6–75.1)].

#### 2.4.2. Peste Des Petits Ruminants (PPR) Small Ruminant Plague

Three studies reported the seroprevalence of PPR (Table S22) and yielded a pooled prevalence of 39.7% [95% CI (30.8–49.4%)].

#### 2.4.3. Rift Valley Fever (RVF)

The disease was reported in cattle in the Northwest and Adamawa regions and small ruminants in the North, while another study gave evidence for the circulation of the disease among livestock in all ten regions of the country (Table S23). The pooled prevalence obtained in small ruminants: 3.7% [95% CI (2.9–4.9%)] (Figure S7) was significantly (*p* = 0.001) lower than that obtained in cattle: 10.9% [95% CI (8.0–14.7%)] (Figure S8).

#### 2.4.4. African Swine Fever (ASF) and Other Viral Diseases of Pigs

African swine fever was reported in pigs in 7 out of 10 regions of Cameroon. Virus isolates were found to belong to three variants of ASF Virus genotype I (Table S24). Risk of introduction of the disease into farms was found to be associated with the entrance of animal health personnel, and locality was the main risk factor identified. A pooled prevalence of 18.9% [95% CI (8.9–35.6%)] was obtained (Figure S9).

#### 2.4.5. Other Viral Diseases of Cattle, Pig, and Poultry

Bovine viral diarrhea, Infectious Bovine Rhinotracheitis (IBR), and Lumpy skin disease were reported in cattle (Table S25). Other viruses identified in pigs included influenza A virus subtype H1N1 in the Centre, North, Far North, and West regions. Hepatitis E was reported in the Littoral, North, West, Centre, and Northwest regions. The zoonotic genotype 3 strain was identified. Porcine hokovirus was reported in the Northwest, Littoral, and Centre regions. Similarly, a novel porcine bocavirus was detected in fecal samples of pigs in several regions of the country. A pooled prevalence of 8.4% [95% CI (2.1–28.0%)] was obtained from five studies on hepatitis E in pigs. Viral diseases of poultry were reported majoritarily (Table S26) after severe outbreaks such as avian Influenza (H5N8, H5N1).

## 3. Discussion

Frequent outbreaks of zoonotic and non-zoonotic diseases as well as dissatisfying productivity levels amongst livestock in Cameroon have prompted several research studies on infectious diseases of animals in the country, providing results that are sparse and limited in space, not covering all regions of the country. Systematic review and meta-analysis procedures used in this study (after quality appraisal of existing papers) are a standard for qualitative and quantitative evidence synthesis, adding value to previous findings [43]. Hence, the quality of publications obtained from the literature were assessed in order to reduce risk of bias.

Therefore, this study showed that most of the studies on infectious diseases of livestock in Cameroon were carried out in the Adamawa region, followed by the West and Northwest regions. Fewer studies were carried out in the East and South regions. This is understandable given that the first three regions are major animal production areas, making up more than 50% of the national cattle headcount and about 70% of the total porcine population of the country [44]. Moreover, the existence of higher education and research institutions in these regions allows availability of researchers and research facilities in these areas [45]. As well, research information was more available for cattle, pigs, and small ruminants than for chicken. This indicates a need for more research studies on diseases of chicken. Microscopy was the most frequently used technique in diagnosing parasitic infections followed by antemortem/postmortem examination, despite being a time consuming and labor-intensive technique [46]; this could be due to its low cost [47]. However, for bacterial diseases, serology was mostly used, closely followed by antemortem/postmortem examination, possibly for reasons similar to those mentioned above. There is a need for increased use of molecular methods as they are more specific and allow for early disease detection [48]. However, the high costs, infrastructure, and technical expertise associated with these methods can limit their adoption and accessibility, making it challenging to adopt and sustain molecular-based infectious disease detection and surveillance programs.

Gastrointestinal parasites and hemoparasites were reported in almost all regions of the country, marking their endemicity. Blood parasites account for a high percentage of cattle deaths and the highest percentage of expenditure incurred by farmers for disease treatment as compared to other diseases [49]. Trypanosomiasis and bovine anaplasmosis were the most prevalent blood cell parasites [14,17,18,24]. The prevalence of trypanosomiasis in cattle was similar to 17.7% obtained by Odeniran and Ademola [50] in Nigeria and different from 8.1% obtained by Leta et al. [51] in Ethiopia. Such similarity with Nigeria may be explained by the fact that both countries are epidemiologically related and share common boundaries, thus facilitating the movement of animals, flies, and consequently pathogen dissemination. This is an indication for the need of an integrated regional effort targeting whole tsetse belts across national borders, as well as the creation of buffer zones, to prevent the reintroduction of tsetse flies and trypanosomes in cleared areas [15,16,19,21]. Moreover, increasing resistance of parasites to trypanocidal drugs hinders chemotherapy-based control efforts [15]. Other mechanical vectors belonging to the families of Tabanidae (tabanid flies) and Muscidae (*Stomoxys*, *Haematobia*, *Haematobosca*) were reported to be responsible for the transmission of trypanosomiasis in Cameroon [52]. This is an indication that the control and prevention strategies to reduce the burden of trypanosomiasis in animal production in Cameroon should be extended to vectors other than tsetse flies. For anaplasmosis, the pooled prevalence was similar to that obtained by Paramanandham et al. [53] in India (11.0%). Meanwhile, the prevalence obtained for babesiosis was higher than 5.3% reported by Haghi et al. [54] in Iran. The high prevalence of anaplasmosis and babesiosis is directly associated with the abundance of ticks, which were also identified in the animal population and environment as well as husbandry systems which are mostly transhumant, favoring infestations [15]. This additionally marks the need for preventive actions and cross-border vigilance, especially with the rapid spread of the dreaded *R. microplus* in neighbouring countries [55,56]. Overall, gastrointestinal parasites showed high prevalence in the different species. Coccidiosis was the most prevalent of all gastrointestinal parasites with a prevalence slightly above the 37% reported by Asfaw et al. [45] in Ethiopia. It causes huge economic losses, mainly in terms of production losses and costs of treatment and prevention [57]. Fascioliasis and paramphistomosis; porcine cysticercosis and monieziosis; and strongyloses and strongyloidiasis were the most identified trematode, cestode, and nematode diseases, respectively. This trend is similar to that observed by Karshima et al. [58] in Nigeria and can be explained by climatic considerations that favor proliferation of snails in the case of trematodes, poor hygiene practices such as lack of latrines that favor porcine cysticercosis, and environmental conditions that are ideal for transmission dynamics of various nematodes [59]. The high prevalence of fasciolosis in the Adamawa region as compared to West and the Northwest regions may be due to the lengthier dry season and consequent scarcity of drinking spots which encourages concentration of animals and hence rapid contamination [44,60]. The wide geographical distribution of these diseases is an indication of the enzootic nature of such pathogens in Cameroon. Moreover, the presence of species with zoonotic potential such as *Fasciola gigantica*, *Oesophagostomum* spp., *Cysticercus cellulosae*, *Strongyloides* spp., *Trichuris* spp., *Trichinella* spp., *Shistosoma* spp., and *Capillaria* spp. presents an indication of obvious public health concerns in Cameroon.

Some of these infections are the main causes of organ condemnation at abattoirs resulting in important economic losses [61]. Alongside ectoparasites, such as scabies, which is the one of the few parasitic diseases listed by law in Cameroon as a disease legally deemed to be contagious (MRLC) was also reported in this study. This calls for enhanced implementation of zoosanitary regulation concerning such diseases.

As for bacterial diseases, pasteurellosis in cattle and salmonellosis in chicken were the most prevalent. The pooled prevalence obtained were similar to those obtained by Asfaw et al. [45] in Ethiopia (51% for salmonella infection in poultry). There is no prophylaxis plan against salmonellosis infection in Cameroon, where it is characterized by high morbidity and mortality rates, in addition to being zoonotic [6]. Other zoonotic bacterial diseases including bovine tuberculosis, brucellosis, and leptospirosis were also reported in several regions of the country with evidence of human contamination [26,28,29,62,63]. The high pooled prevalence obtained for these diseases listed as contagious notified diseases (“*Maladies Réputée Légalement Contagieuses*,” MRLCs) indicates a possible low level of implementation of regulation and prevention/control strategies at the national level [32,33,34]. The surveillance plans for the majority of these infections within the Cameroon Animal Disease Epidemiology Surveillance Network (RESCAM) are generally delayed by factors such as limited resources. There is, therefore, a need for reinforcement of financial, human, and technical capacities for better surveillance [12]. There were few reports concerning diseases such as Contagious Bovine Pleuropneumonia anthrax, pasteurellosis, Q fever, and abortive diseases, though some of these diseases are regularly reported to the surveillance systems and of even higher interest for the Ministry in charge of livestock and veterinary services [64]. Hence, there is need for research on their epidemiology in the country, in order to provide information for efficient prevention and control strategies. Very few studies reported bacterial diseases in small ruminants and pigs. Meanwhile, bacterial diseases of pigs such as salmonellosis, colibacillosis, pasteurellosis, and erysipelas are known to exist in the country causing heavy losses every year [65]. Likewise, bacterial diseases of small ruminants like salmonellosis, Contagious Caprine Pleuropneumonia, pasteurellosis, tuberculosis, and abortive diseases, which are OIE-listed diseases (World Organization for Animal Health), are also known to be present but knowledge of their impact is scarce [66]. Hence, studies should be conducted to determine the exact burden of these diseases in Cameroon.

This study highlights frequent outbreaks of highly pathogenic viral infections in the country such as avian influenza and African swine fever virus. Such diseases generally result in severe market shocks when consumers fear that animal products or exposure in markets will make them ill. This can lead to a sharp fall in consumption during outbreak, leading to a fall in prices and loss of revenue for producers until consumer confidence is restored [67]. This calls for improvement of biosecurity measures at the level of national frontiers and individual farms. *Peste des Petits Ruminants* and Foot-and-mouth disease [36,37,38,39] were the most prevalent infections with values higher than those obtained by Barman et al. [43] in Northeastern India (15.0% for *Peste des Petits Ruminants* and 21.0% for Foot-and-mouth disease in cattle). Other diseases of zoonotic importance such as Rift Valley Fever, porcine hepatitis E virus, and Influenza H1N1 were found to be enzootic in the country. Rift Valley Fever and Foot-and-mouth disease are at the origin of trade restrictions, thus limiting the profit that would have been made from exports [38,68]. The detection of novel viruses such as porcine bocaviruses and hokoviruses reflects the impact of globalization on the distribution of new and emerging viruses and is a call to intensify actions to prevent entrance of such diseases into the country. The high prevalence reports for scarcely reported diseases such as Infectious Bovine Rhinotracheitis and other infections including Bovine viral diarrhea, chicken anemia virus, and Newcastle’s disease call for more research to be done concerning the epidemiology of those diseases.

Overall, there was marked heterogeneity amongst studies that were included in most of the meta-analyses (80%). Prevalence values varied greatly between studies included in the same analyses. Such heterogeneity could be accounted for by varying sensitivity and specificity of the diagnostic techniques and study sites (regions). Publication bias was significant in few analyses (5.7%) and may have been influenced by the exclusion of some studies from the analysis because of incomplete information. Even so, such a low frequency of publication bias implies that the pooled estimates obtained in this study are an acceptable reflection of the real situation in the country. Hence, the results of this study will be useful in guiding informed decision-making for the establishment of efficient control programs against infectious diseases of livestock in Cameroon, in a bid to reduce economic losses and public health problems associated with these diseases. It should be noted, however, that data included in this study was obtained mainly from published articles and unpublished dissertations and did not include notification data from the epidemiological surveillance network of the country. Based on disease prevalence, the most prioritized major infectious diseases of livestock in Cameroon should be gastrointestinal parasitosis in all species, trypanosomiasis, bovine pasteurellosis, avian salmonellosis, small ruminant plague, foot-and-mouth disease, and African swine fever. Other important endemic zoonoses in the country were Rift Valley fever, bovine tuberculosis, and brucellosis. These diseases are also priority of the national epidemiological surveillance network [12]. This study does not only highlight viral and bacterial diseases as being the most important as in several prioritization exercises but also underlines the burden of parasitic diseases which are often more important from farmers’ perspectives [11]. It also means that compromises should be found when addressing those viral and bacterial diseases identified as national priorities by including the control of parasitic diseases at farm level in the form of health packages that are more likely to enhance farmers’ engagement, one of the keys to any successful disease detection and response program. The data from the survey and the notification should therefore be used in a complementary way as a possible first important step in the process of prioritizing animal diseases, including zoonosis. This will allow a better implementation of pillars 1 and 3 of Cameroon’s Animal Health and Veterinary Public Health Policy.

## 4. Materials and Methods

### 4.1. Type, Period, and Area of Study

The study consisted of a systematic review of literature reporting major infectious diseases in the main food-producing species (cattle, pigs, small ruminants, and poultry) in Cameroon and a meta-analysis of their prevalence. The term “infectious disease” as used in the context of this work applies to parasitic, bacterial, and viral diseases following the definition given by the World health Organization. This study used secondary data and therefore ethical clearance was not required. 

### 4.2. Search Strategy

A systematic review was conducted according to PRISMA (Preferred Reporting Items for Systematic Reviews and Meta-Analyses) guidelines [69]. Records were retrieved between November 2019 and March 2020 from three web databases, namely PubMed, African Journals online (AJOL), and Google scholar for articles published in French and English between 2000 and 2020. A search string with specific keywords for each database was developed and the PRISMA checklist was also used (Supplementary S2). Grey literature comprising theses were retrieved by onsite consultation at the libraries of the main higher education institutes training in animal health and husbandry. Reference lists of relevant articles were also screened for additional titles to be included in the review. National subject matter experts were contacted for additional articles. Full-text records on the prevalence, incidence, epidemiology, and/or risk factors of infectious diseases of target food-producing animals in Cameroon were included in the review. Only records published between January 2000 and March 2020 in French or English were taken into consideration. Search results were also limited to observational (cross-sectional or longitudinal) and non-randomized case-control studies conducted on target food-producing animals. Publications that did not respect the criteria listed above as well as those reporting randomized control trials were excluded from the review. A randomized controlled trial is performed under controlled conditions with random allocation of interventions to comparison groups to determine whether a cause–effect relation exists between an intervention and an outcome. This was irrelevant to the present study. Additional criteria were used to evaluate studies that were fit for meta-analysis. Studies were included in the meta-analysis if they reported the number of positive samples for the particular livestock diseases, the number of animals that had been tested, and the standard test used for diagnosis. Meta-analysis was done for diseases for which at least two studies met the aforementioned criteria [70].

### 4.3. Article Quality Assessment

Quality assessment of the primary studies was done to evaluate the reliability of studies using a modified version of a critical appraisal tool developed for use in systematic reviews addressing questions of prevalence [71]. Each publication was assessed using 8 specific questions (Supplementary S3) and the responses to each question were coded as “yes,” “no,” “irrelevant,” or “unclear”. They were then categorized into three groups: articles that had more than 75% of items answered “yes” were classified as “High quality” (H), articles that had between 50% and 75% of items answered “yes” were considered of “Medium quality” (M), and articles with less than 50% of items answered “yes” were considered of “Low quality” (L). Only “High quality” articles were included in the review as they showed low risk of bias.

### 4.4. Data Extraction and Analysis

Data were extracted from records that met the inclusion criteria and passed quality assessment. The information collected included publication information, disease condition (disease type, evidence of zoonotic potential), study design, and results (prevalence, risk factors). Descriptive statistics were carried out to characterize primary articles included in the study. The point estimate prevalence and 95% confidence interval (CI) for each disease and specific animal species pair were pooled using a random-effects model. Heterogeneity across the studies was assessed using the Cochrane Q statistics and was quantified with the I^2^ statistic [72]. The Begg rank correlation [73] and Egger regression asymmetry test [74] were used for publication bias analysis. If publication bias was confirmed, the trim-and-fill funnel plot-based model developed by Duval and Tweedie [75] was used to adjust for the bias. Subgroup analysis was performed according to the region for diseases that were reported in three or more different regions. Chi-square test was used to compare the pooled regional prevalence obtained. Meta-analysis was run separately for different parasitic, bacterial, and viral diseases paired with each animal species. A *p*-value of 0.05 was considered statistically significant, except for the test of heterogeneity which served only to estimate the magnitude of the true dispersion of the studies effect size [76]. The meta-analysis was conducted using Comprehensive Meta-Analysis Software (Biostat, Inc., Hoboken, NJ, USA) Version 3.0 for Windows.

## 5. Conclusions

This study highlights the distribution and prevalence of various bacterial, parasitic, and viral infections of pigs, cattle, small ruminants, and chicken in Cameroon based on published and grey literature after quality appraisal. Several diseases of livestock have been reported in Cameroon with high prevalence and evidence for the endemicity of major zoonotic infections in Cameroon warrants for enhanced public health interventions strategically targeting prioritized animal diseases for surveillance and control. Overall, there is dearth information concerning some infectious livestock diseases in Cameroon and the research is concentrated to some regions. There is need for research to be done on their epidemiology in the country, to provide information for efficient prevention and control strategies. This baseline data collected on infectious diseases can be a first step for national disease prioritization and can support the identification of dominant pathologies for livestock to define and adjust effective prevention and control measures to be implemented at (sub-) national level.

## Figures and Tables

**Table 1:** General characteristics and distribution of studies included in the review.

Characteristics	Number of Studies on Parasitic Diseases N (%)	Number of Studies on Bacterial Diseases N (%)	Number of Studies on Viral Diseases N (%)	Total N (%)
Region				
Adamawa (AD)	30 (31.91)	14 (35.90)	13 (36.11)	57 (34.54)
Centre (C)	5 (5.32)	6 (15.38)	8 (22.22)	19 (11.51)
East (E)	3 (3.19)	2 (5.13)	2 (5.55)	7 (4.24)
Far North (FN)	5 (5.32)	5 (12.8)	6 (16.67)	16 (9.69)
Littoral (L)	3 (3.19)	4 (10.25)	3 (8.33)	10 (6.06)
North (N)	15 (15.95)	6 (15.38)	10 (27.77)	31 (18.78)
Northwest (NW)	13 (13.83)	12 (30.76)	5 (13.88)	30 (18.18)
South (S)	2 (2.12)	0 (0)	3 (8.33)	5 (3.03)
Southwest (SW)	12 (12.76)	1 (2.56)	2 (5.55)	15 (9.09)
West (W)	20 (21.27)	6 (15.38)	8 (22.22)	34 (20.60)
Study site not precise	1 (1.06)	0 (0)	4 (11.11)	5 (3.03)
Publication period				
2000–2004	8 (8.51)	1 (2.56)	2 (5.55)	11 (6.66)
2005–2009	10 (10.64)	4 (10.25)	1 (2.77)	15 (9.09)
2010–2014	19 (20.21)	13 (33.33)	12 (33.33)	44 (26.66)
2015–March 2020	57 (60.64)	21 (53.84)	21 (58.33)	95 (57.58)
Species				
Chicken	7 (7.44)	5 (12.82)	5 (13.89)	17 (10.30)
Pigs	24 (25.53)	2 (5.12)	12 (33.33)	37 (22.42)
Cattle	50 (53.19)	29 (74.35)	14 (38.88)	90 (54.54)
Sheep	14 (14.89)	1 (2.56)	7 (19.44)	22 (13.33)
Goats	15 (15.95)	3 (7.69)	6 (16.66)	24 (14.54)
Type of material				
Journal article	56 (59.57)	25 (64.10)	22 (61.11)	101 (61.21)
Dissertation (unpublished)	38 (40.43)	14 (35.90)	14 (38.89)	64 (38.79)
Language				
English	53 (56.38)	25 (64.10)	21 (58.33)	99 (60)
French	41 (43.62)	14 (35.90)	15 (41.67)	66 (40)
Study design				
Cross-sectional	92 (97.87)	37 (94.87)	30 (83.33)	155 (93.94)
Longitudinal	2 (2.13)	2 (5.13)	6 (16.67)	10 (6.06)
Samples				
Blood	42 (44.68)	17 (43.58)	21 (58.33)	80 (48.48)
Fecal or rectal swab	29 (30.85)	5 (12.82)	1 (2.77)	35 (21.21)
Meat or carcass	16 (17.02)	12 (30.76)	4 (11.11)	36 (21.81)
Skin	8 (8.51)	4 (10.25)	1(2.77)	13 (7.87)
Others (milk, uterine swabs…)	0 (0)	4 (10.25)	4 (11.11)	8 (4.84)
Diagnostic methods				
Serology (ELISA, RBPT, VN)	9 (9.57)	16 (41.02)	16 (44.44)	41 (24.26)
Molecular (PCR, sequencing)	11 (11.70)	1 (2.56)	14 (38.88)	26 (15.38)
PM/AM examination	18 (19.15)	11 (28.2)	4 (11.11)	33 (19.53)
Microscopy	53 (56.38)	8 (20.51)	/	61 (36.10)
Others	3 (3.19)	3 (7.69)	2 (5.55)	8 (4.73)
Total	94 (56.97)	39 (23.64)	36 (21.82)	169 (100)

Legend: ELISA: Enzyme-Linked Immunosorbent Assay; RBPT: Rose Bengal Plate Test; VN: Virus Neutralization; PCR: Polymerase Chain Reaction; PM: Postmortem; AM: Antemortem; TST: Tuberculin Skin Test.

**Table 2:** Summary of meta-analysis results of parasitic diseases of food-producing animals in Cameroon.

Disease Studied	Species	N° of Study Results	Total Number of Samples	N° of Positive Samples	Meta-Analysis Data						
					Model	Pooled Prevalence (%)	95% CI	Tau^2^	I^2^ (%)	*p*-Value	Relative Weight Range (%)
Trypanosomiasis	Cattle	18	7369	1641	Random	20.9	14.9–28.7	0.789	93.514	0.000	5.12–5.66
Trypanosomiasis	Goats	5	963	270	Random	28.4	12.7–51.9	1.233	95.934	0.000	18.74–20.84
Trypanosomiasis	Pigs	6	1491	551	Random	54.2	39.6–68.2	0.507	95.169	0.000	14.12–17.51
Trypanosomiasis	Sheep	5	657	185	Random	28.4	12.7–51.9	1.233	95.934	0.000	18.74–20.84
Babesiosis	Small ruminants	4	1081	35	Random	3.5	2.2–5.6	0.119	47.519	0.167	18.87–30.87
Babesiosis	Cattle	2	925	208	Random	21.6	4.9–59.7	1.455	98.956	0.000	49.85–50.15
Theileriosis	Small ruminants	5	1478	42	Random	4.9	1.9–11.8	1.079	96.164	0.000	18.49–21.45
Anaplasmosis	small ruminants	5	1478	189	Random	13.0	6.2–25.2	0.816	97.792	0.000	19.32–20.56
Anaplasmosis	Cattle	2	925	324	Random	35.1	4.7–85.5	2.951	99.546	0.000	49.96–50.04
Gastrointestinal parasites	Cattle	6	1690	989	Random	57.4	45.3–68.7	0.354	95.622	0.000	16.34–17.06
Gastrointestinal parasites	Poultry	3	1701	1076	Random	67.2	48.3–81.8	0.469	88.632	0.000	32.87–33.78
Gastrointestinal parasites	Pigs	3	741	638	Random	88.0	78.2–93.8	0.328	97.988	0.000	24.66–37.79
Fascioliasis	Cattle	16	18,510	3581	Random	17.3	11.0–26.2	1.114	99.39	0.000	5.46–6.47
Fascioliasis	Small Ruminants	3	1085	110	Random	8.8	2.1–30.2	1.710	97.727	0.000	19.38–58.21
Paramphistomosis	Cattle	4	1415	228	Random	12.6	4.5–30.8	1.251	97.834	0.000	21.98–26.11
Paramphistomosis	Goats	2	460	29	Random	19.7	3.2–64.7	2.982	98.463	0.000	49.48–50.52
Paramphistomosis	Pigs	2	495	171	Random	23.0	5.4–61.0	1.373	96.056	0.000	48.39–51.61
Cysticercosis	Pigs	10	7553	348	Random	6.2	3.3–11.3	0.940	96.092	0.000	3.8–11.74
Coccidiosis	Poultry	4	2581	1276	Random	49.2	28.0–70.7	0.859	99.107	0.000	24.86–25.12
Coccidiosis	Cattle	2	660	43	Random	4.5	1.1–17.0	1.003	87.628	0.004	45.05–54.96
Coccidiosis	Goats	2	349	122	Random	55.1	2.8–97.5	0.000	99.103	0.000	49.79–50.21
Coccidiosis	Pigs	4	1677	521	Random	30.2	10.3–61.9	2.157	98.983	0.000	24.92–25.10
Monieziosis	Cattle	3	1015	36	Random	3.6	2.4–5.4	0.044	30.120	0.239	12.29–46.27
*Strongylus* spp.	Cattle	5	1244	552	Random	34.5	17.2–57.2	1.097	98.019	0.000	19.71–20.34
*Strongylus* spp.	Pigs	4	1419	677	Random	48.6	28.6–69.1	0.750	98.182	0.000	24.23–25.35
*Trichuris* spp.	Cattle	2	555	44	Random	3.0	0.1–43.6	4.967	90.594	0.001	45.54–54.46
*Trichuris* spp.	Pigs	4	1281	81	Random	4.4	0.8–20.9	2.997	96.818	0.000	23.58–26.04
*Strongyloides* spp.	Pigs	5	1677	326	Random	22.0	14.1–32.6	0.358	94.502	0.000	19.41–20.55
*Strongyloides* spp.	Cattle	4	1104	121	Random	12.4	3.6–35.1	1.834	97.581	0.000	24.72–25.23
*Strongyloides* spp.	Sheep	2	303	30	Random	7.9	0.6–56.8	3.734	96.058	0.000	48.69–51.31
*Strongyloides* spp.	Goats	3	554	63	Random	10.0	1.0–54.3	4.283	97.949	0.000	32.82–33.79
*Strongyloides* spp.	Poultry	2	1272	108	Random	8.5	7.1–10.2	0.000	0.000	0.909	29.6–70.33
*Ascaridia* spp.	Poultry	3	1701	360	Random	22.9	11.8–39.7	0.488	97.722	0.000	33.14–33.49
*Heterakis* spp.	Poultry	3	1701	295	Random	18.4	7.3–39.5	0.865	98.442	0.000	33.18–33.44
*Capillaria* spp.	Poultry	2	1318	109	Random	8.9	3.7–19.7	0.425	95.423	0.000	49.95–50.05

Legend: CI: Confidence interval; I^2^: Inverse variance index; Tau^2^: the tau-squared is the between-study variance.

**Table 3:** Summary of meta-analysis results of bacterial diseases of food-producing animals in Cameroon.

Disease Studied	Species	N° of Study Results	Total Number of Samples	N° of Positive Samples	Meta-Analysis Data						
					Model	Pooled Prevalence (%)	95% CI	Tau^2^	I^2^ (%)	*p*-Value	Relative Weight Range (%)
Tuberculosis	Cattle	15	121,612	1588	random	3.3	1.5–6.9	2.395	99.724	0.000	6.45–6.73
Brucellosis	Cattle	10	6776	436	Random	7.0	4.8–10.0	0.372	93.514	0.000	8.10–10.55
Brucellosis	Pigs	2	1081	20	Random	8.0	0.0–17.40	4.778	82.309	0.017	41.60–58.40
Salmonellosis	Poultry	3	484	343	Random	48.2	12.9–65.5	2.359	97.299	0.000	26.16–37.02
Leptospirosis	Cattle	2	1549	35	Random	2.5	0.0–64.2	9.202	98.448	0.000	49.43–50.57
Pasteurellosis	Cattle	3	362	198	Random	55.5	39.2–70.7	0.292	87.934	0.000	29.20–36.02

Legend: CI: Confidence interval; I^2^: Inverse variance index; Tau^2^: the tau-squared is the between-study variance.

**Table 4:** Summary of meta-analysis results of various viral diseases of food-producing animals in Cameroon.

Disease Studied	Species	N° of Study Results	Total Number of Samples	N° of Positive Samples	Meta-Analysis Data						
					Model	Pooled Prevalence (%)	95% CI	Tau^2^	I^2^ (%)	*p*-Value	Relative Weight Range (%)
Foot-and-Mouth Disease	Cattle	9	19,778	4897	Random	39.4	20.4–62.4	2.000	99.679	0.000	11.03–11.16
	Sheep	3	1251	128	Random	8.1	3.6–17.4	0.551	93.194	0.000	32.66–34.30
	Pigs	2	658	29	Random	4.4	3.1–6.3	0.000	0.000	0.710	6.97–93.03
*Peste des Petits Ruminants*	Small ruminants	4	2090	791	Random	39.7	30.8–49.4	0.133	88.768	0.000	18.81–29.41
Rift Valley Fever	Small ruminants	10	1525	53	Random	3.7	2.9–4.9	0.000	0.000	0.566	1.82–28.12
	Cattle	7	2532	256	Random	10.9	8.0–14.7	0.149	81.722	0.000	2.47–18.62
African Swine Fever	Pigs	6	2472	643	Random	18.9	8.9–35.6	1.117	98.150	0.000	15.32–17.37
Hepatitis E	Pigs	5	507	73	Random	8.4	2.1–28.0	2.114	92.862	0.000	13.06–24.74

Legend: CI: Confidence interval; I^2^: Inverse variance index; Tau^2^: the tau-squared is the between-study variance.

## Data Availability

The data that support the findings of this study are available on request from the corresponding author.

## Supplementary Materials

The following supporting information can be downloaded at: https://www.mdpi.com/article/10.3390/pathogens12091076/s1, Supplementary Material S1. Figure S1. Forest plot of pooled prevalence of trypanosomiasis in pigs; Figure S2. Forest plot of pooled prevalence of trypanosomiasis in small ruminants; Figure S3. Forest plot of pooled prevalence of gastro intestinal parasites in cattle in Cameroon; Figure S4. Forest plot of pooled prevalence of fascioliasis in cattle in Cameroon; Figure S5. Forest plot of pooled prevalence of porcine cysticercosis in Cameroon; Figure S6. Forest plot of pooled prevalence of brucellosis in cattle in Cameroon; Figure S7. Forest plot of pooled prevalence of RVF in small ruminants in Cameroon; Figure S8. Forest plot of pooled prevalence of RVF in cattle in Cameroon; Figure S9. Forest plot of pooled prevalence of African swine fever in pigs in Cameroon; Table S1. Characteristic of studies reported the outcome of Trypanosomiasis infections in animal in Cameroon; Table S2. Pooled regional prevalences of trypanosomiasis in cattle; Table S3. Characteristics and distribution of studies reporting other hemoparasitic infections in animals in Cameroon; Table S4. Pooled prevalence of tick-borne parasites in ruminants in Cameroon; Table S5. Characteristics and distribution of studies reporting gastrointestinal parasites in animals in Cameroon; Table S6. Characteristics and distribution of studies reporting fascioliasis in Cameroon; Table S7. Pooled regional prevalences of fascioliasis in cattle in Cameroon; Table S8. Characteristics and distribution of studies reporting trematodes other than fascioliasis in animals in Cameroon; Table S9. Characteristics and distribution of studies reporting cysticercosis in Cameroon; Table S10. Pooled regional prevalence of porcine cysticercosis in Cameroon; Table S11. Characteristics and distribution of studies reporting cestode diseases other than cysticercosis in Cameroon; Table S12. Characteristics and distribution of studies reporting nematodes in animals in Cameroon; Table S13. Characteristics and distribution of studies reporting coccidiosis in animals in Cameroon; Table S14. Species of ticks reported in Cameroon; Table S15. Characteristics and distribution of studies reporting brucellosis in animals in Cameroon; Table S16. Regional pooled prevalence of brucellosis in cattle in Cameroon; Table S17. Characteristics and distribution of studies reporting tuberculosis in animals in Cameroon; Table S18. Pooled regional prevalence of bovine tuberculosis; Table S19. Characteristics and distribution of studies reporting salmonellosis, colibacillosis and campylobacteriosis in animals in Cameroon; Table S20. Characteristics and distribution of studies reporting other bacterial diseases in animals in Cameroon; Table S21. Characteristics and distributions of studies reporting FMD in animals in Cameroon; Table S22. Characteristics and distribution of studies reporting SRP in small ruminants in Cameroon; Table S23. Characteristics and distribution of studies reporting RVF in Cameroon; Table S24. Characteristics and distribution of studies reporting ASF and other viral diseases of pigs in Cameroon; Table S25. Characteristics and distribution of studies reporting other viral diseases of cattle in Cameroon; Table S26. Characteristics and distribution of studies reporting viral diseases of poultry in Cameroon. Supplementary Material S2. Prisma check list used; Supplementary Material S3. Study quality assessment form and decision.
