# Supplementary material for: Zoo-Sanitary Situation Assessment, an Initial Step in Country Disease Prioritization Process: Systematic Review and Meta-Analysis from 2000 to 2020 in Cameroon
Source: Pathogens. 2023 Aug 24;12(9):1076. doi: 10.3390/pathogens12091076 (PMC10537522; doi:10.3390/pathogens12091076)
Supplement: Supplementary file 1 [file pathogens-12-01076-s001.zip › pathogens-2538444-supplementary.pdf]

Supplementary materials

Supplementary S1. Tables

Table S1. Characteristic of studies reported the outcome of Trypanosomiasis infections in animal in Cameroon

	Cattle	Pigs	Small ruminants	Total
Number of studies	17[a]	7[b]	5[c]	24
Regions				
Adamawa	9	1	1	11
North	7	/	/	7
Far North	1	/	/	1
East	1	/	/	1
North West	1	/	/	1
South West	/	6	4	6
Species identified				
T. congolense	17	3	4	24
T. vivax	17	2	2	21
T. brucei	16	4	2	22
T. theileri	2	/	/	2
T. grayi	2	/	/	2
T simaie	/	1	1	1
Samples used				
Blood	17	7	5	24
Mid-gut	/	/	/	2
Diagnostic tests				
PCR	2	4	3	6
Microscopy	14	1	1	15
ELISA	1	/	/	1
parasite culture	/	1	/	1
Card Agglutination test	/	1	1	1
Risk factors				
Body condition score	1	/	/	1
Breed	1	/	/	1
production systems	1	/	/	1
prophylactic measures	1	/	/	1
Age	1	/	1	2
Sex	2	/	1	3
Season	1	/	1	2

Legend: [a]: (Awa and Ndamkou, 2006; Mamoudou et al., 2006a; Mamoudou et al., 2006b; Achukwi and Musongong, 2009; Mamoudou et al., 2009; Tanenbe et al., 2010; Mpouam et al., 2011; Mamoudou et al., 2015a; Mamoudou et al., 2015b; Mamoudou et al., 2016a; Mamoudou et al., 2016c; Ikoum, 2017; Mohamadou, 2017; Ngomtcho et al., 2017; Soussai, 2017; Paguem et al., 2019); [b]: (Nkinin et al., 2002; Simo et al., 2006; Nimpaye et al., 2011; Simo et al., 2012; Simo et al., 2013; Deya-yang, 2016; Fogue et al., 2017) ; [c]: (Nimpaye et al., 2011; Simo et al., 2012; Simo et al., 2013; Diezoumbe, 2017; Fogue et al., 2017).

Table S2. Pooled regional prevalences of trypanosomiasis in cattle

Region	Number of study reports included in meta-analysis	Pooled prevalence (95%CI)	P-value
Adamawa	9	26.1 (18.6-35.3)	0.41
North	6	16.8 (7.5-33.6)	

Table S3. Characteristics and distribution of studies reporting other hemoparasitic infections in animals in Cameroon

Characteristics		Cattle	Small ruminants	Pigs	Chicken	Total
Number of studies		4[a]	3[b]	1[c]	1[d]	9
Region						
Adamawa		3	1	1	/	5
East		/	/	/	1	1
Far North		1	/	/	/	1
South		/	1	/	/	1
North		1	1	/	/	2
North West		1	/	/	/	1
Species identified						
Babesia	*Bovis*	3	2	1	/	6
	Bigemina	3	2	1	/	6
Theileria		1	3	/	/	4
	Centrale	3	2	1	/	7
Anaplasma	*Marginale*	3	2	1	/	7
	Ovis	/	1	/	/	1
Borrelia spp		1	/	/	/	1
Ricketssia spp		1	/	/	/	1
Erlichia spp		1	/	/	/	1
Leucocytozoon schoutedeni		/	/	/	1	1
Risk factors						
Age		/	1	/	/	1
Sex		/	2	/	/	2
Season		/	1	/	/	1
Samples		Blood				
Diagnostic techniques BCT		1	/	1	/	2
Blood smear		1	1	/	/	1
GIEMSIA staining		2	2	/	/	3
PCR			/	/	1	3

Legend : [a]: (Esemu et al., 2014; Mamoudou et al., 2017; Soussai, 2017; Abanda et al., 2019) ; [b]: (An'naguil, 2017; Diezoumbe, 2017; Dongmo, 2017a) ; [c]: (Deya-yang, 2016) ; [d]: (Sehgal et al., 2006).

Table S4. Pooled prevalence of tick-borne parasites in ruminants in Cameroon

**Disease agent**	**Species**	**Number of studie**	**Pooled prevalence (95% (**	**P**	**I²**
Babesia spp	Small ruminants	2	3.5 (2.2-5.6)	0.167	47.519
	Cattle	2	21.6 (4.9-59.7)	0.000	98.956
Theileria spp	Small ruminants	3	4.9 (1.9-11.8)	0.000	96.164

Anaplasma spp	small ruminants	3	10.9 (3.8-27.8)	0.000	97.792
	Cattle	2	35.1 (4.7-85.5)	0.000	99.546

Table S5. Characteristics and distribution of studies reporting gastrointestinal parasites in animals in Cameroon

Characteristics*	Cattle	Sheep	Goats	Pigs	Chicken	Total
Number of studies	10	3	5	5	3	22
Region						
Adamawa	4	1	1	1	1	7
Centre	/	/	/	1	/	1
Far North	/	/	/	1	/	1
Littoral	/	/	1	/	/	1
North	2	/	/	/	/	2
North West	2	1	1	/	/	2
South west	1	/	/	/	1	6
West	1	1	2	2	1	5
Samples	Faeces					
Diagnostic techniques	Coprology					
Risk factors						
Season	1	/	/	/	/	1
Age	1	1	2	2	/	5
Farm hygiene	/	/	/	2	/	2
Species	/	1	1	/	/	1
Sex	1	1	2	/	/	3
Locality	1	/	/	/	1	2
Feeding	/	/	/	1	1	2
Prophylaxis	1	/	/	/	1	2
Farmer's knowledge of the disease	/	/	/	/	1	1

Legend: *: results do not include studies that reported fascioliasis or cysticercosis only.

Table S6. Characteristics and distribution of studies reporting fascioliasis in Cameroon

Characteristics	Cattle	Sheep	Goats	Pigs	Total
Number of studies	15[a]	2[b]	1[c]	1[d]	19
Regions					
North west	3	/	/	/	3
Adamawa	8	1	1	1	10
Centre	1	1	/	/	1
South West	1	/	/	/	1
Littoral	1	/	/	/	1
West	3	/	/	/	3
Samples					
Blood	1	/	/	/	1
Faeces	8	1	1	1	10
Live animal or carcass	8	1	/	/	9
Diagnostic techniques					
ELISA	1	/	/	1	1
Coprology	9	1	1	/	11
AM/PM	7	1	/	/	8

Fasciola species					
Fasciola hepatica	2	/	/	/	2
Fasciola gigantica	13	2	1	1	17

Legend: [a]: (Tebug, 2009; Abakar, 2014; Fotso, 2014; Nzale, 2014; Dicko, 2015; Manchang et al., 2016; Salhine, 2016; Tchounkeu, 2017; Ekoual, 2018; Kelly et al., 2018; Kouam et al., 2018a; Mbotake, 2019; Takang et al., 2019; Yemelong, 2019; Chahdini et al., 2020); [b]: (Anoumbo, 2017; Takang et al., 2019); [c]: (Anoumbo, 2017)
d: (Deya-yang, 2016).

Table S7. Pooled regional prevalences of fascioliasis in cattle in Cameroon

Region	Number of study reports included in meta-analysis	Pooled prevalence (95%CI)	P-value
Adamawa	7	41.5 (27.9-56.6)	0.006
North West	3	2.4 (0.9-6.4)	
West	3	11.5 (5.0-24.4)	

Table S8. Characteristics and distribution of studies reporting trematodes other than fascioliasis in animals in Cameroon

Characteristics			Cattle	Small ruminants	Pigs	poultry	Total
		Number of studies	7[a]	2[b]	1[c]	1[d]	10
Species Identified		Region					
	Paramphistomum spp	AD	2	1	1	/	4
		W	1		/	/	1
		NW	1		/	/	1
		L	/	1	/	/	1
	Echinostoma spp	AD	/	/	/	1	1
	Schistosoma bovis	AD	1	/	/	/	1
	Dicrocoelium hopes	AD	2	/	/	/	2
Samples Used	Faeces						
Diagnostic methods	Coprology						

Legend: [a]: (Dicko, 2015; Mamoudou et al., 2016d; Tchounkeu, 2017; Ekoual, 2018; Djuikwo-Teukeng et al., 2019; Mbotake, 2019; Chahdini et al., 2020); [b]: (Simeni, 2014; Anoumbo, 2017); [c]: (Deya-yang, 2016); [d]: (Bagari, 2019).

Table S9. Characteristics and distribution of studies reporting cysticercosis in Cameroon

Characteristics	Pigs	Cattle	Small ruminants	Total
	10[a]	1[b]	1[c]	12

Region				
Adamawa	/	1	/	1
Centre	1	/	/	1
North West	1	/	/	1
North	3	/	1	4
West	4	/	/	4
Whole territory	1	/	/	1
Samples				
Blood	5	1	1	6
Carcass	4			5
Diagnostic techniques				
ELISA	5	/	1	6
PM	4	1	/	5
Risk factors				
Age of pigs	1	/	/	1
Rearing system	2	/	/	2
Presence of latrine	2	/	/	2
Feeding	1	/	/	1
Farmer's knowledge of the disease	2	/	/	2
Farm hygiene	3	/	/	3

Legnd: [a]: (Azebaze, 2000; Tchinda, 2000; Assana et al., 2001; Pouedet et al., 2002; Praet et al., 2009; Assana et al., 2010; Ngwing et al., 2012; Assana et al., 2019a; Assana et al., 2019b; Dongmo, 2019); [b]: (Manchang et al., 2016); [c]: (Djonmaila, 2016).

Table S10. Pooled regional prevalence of porcine cysticercosis in Cameroon

Region	Number of study reports included in meta-analysis	Pooled prevalence (95%CI)	P-value
West	4	3.2 (1.0-9.6)	0.25
North	3	21.5 (9.9-40.6)	
Centre	2	8.75 (6.3-12.6)	

Table S11. Characteristics and distribution of studies reporting cestode diseases other than cysticercosis in Cameroon

Disease or disease agent	Characteristics	Cattle	Small ruminants	Chicken	Total
Monieziosis	Number of studies	4[a]	3[b]	1[c]	7
	Region				
	Adamawa	1	2	/	1
	West	1	1	/	3
	South West	1	/	/	1
	Littoral	/	/	/	1
	North West	1	/	/	1
	Samples	Faeces			
	Diagnostic techniques	Coprology			
	Region				
	Adamawa	/	/	1	1

Choanotenia spp, *Amoebotenia* spp, *Hymenolepis* spp, *Railiettina* spp	Sample	/	/	Faeces	
	Diagnostic technique	/	/	Coprology	
Muscular and cerebral coenuruses	Region				
	North	/	1	/	1
	Samples	/	Tissue	/	
	Diagnostic technique	/	Optical microscopy	/	

Legend: [a]: (Dicko, 2015; Mamoudou et al., 2016d; Kouam et al., 2018a; Mbotake, 2019); [b]: (Simeni, 2014; Tchouagoue, 2014; Djonmaila, 2016; Mbiakop, 2018); [c]: (Bagari, 2019).

Table S12. Characteristics and distribution of studies reporting nematodes in animals in Cameroon

Characteristics	Cattle	Sheep	Goats	Pigs	Chicken	Total
Number of studies	11[a]	3[b]	5[c]	7[d]	3[e]	25
Region						
Adamawa	5	1	1	1	1	8
Centre	/	/	/	1	/	1
Far North	/	/	/	2	/	2
Littoral	/	/	1	/	/	1
North	2	/	/	/	/	2
North West	2	1	1	/	1	2
South West	1	/	/	/	1	2
West	1	1	2	4	/	7
Species identified						
Strongylida						
Trichostrongylus spp.	2	2	2	/	1	4
Ostertagia spp.	2	1	1	/	/	2
Hyostrongylus rubidium	/	/	/	/	2	2
Haemonchus spp.	2	1	1	/	/	2
Cooperia spp.	1	/	/	/	/	1
Nematodirus spp.	1	/	/	/	/	1
Strongylus spp.	7	1	4	3	/	14
Oesophagostomum spp.	2	1	1	2	/	4
Bunostomum spp.	1	/	/	/	/	1
Metastrongylus spp.	/	/	/	/	1	1
Rhabditoidea						
Strongyloides spp.	6	3	6	5	2	20
Ascaridoidea						
Ascaris spp.	/	/	/	/	3	3
Toxocara spp.	3	/	/	1	/	4
Ascaridia spp.	/	/	/	/	3	3
Heterakis spp.	/	/	/	/	3	3
Spirurida						
Gongylonema spp	/	/	/	/	1	1
Tetrameres spp.	/	/	/	/	1	1
Onchocerca ochengi	1	/	/	/	/	1

Enoplida						
Trichuris spp.	3	2	5	4	2	13
Capillaria spp.	/	/	/	/	2	2
Trichinella spp.	1	/	/	1	/	1

Legend: [a]: (Chollet et al., 2000; Ntonifor et al., 2013; Dicko, 2015; Mamoudou et al., 2015c; Kouam et al., 2018a; Mbotake, 2019); [b]: (Ntonifor et al., 2013; Anoumbo, 2017; Mbiakop, 2018); [c]: (Chollet et al., 2000; Ntonifor et al., 2013; Simeni, 2014; Tchouagoue, 2014; Mamoudou et al., 2015c; Anoumbo, 2017; Mbiakop, 2018); [d]: (Byambas, 2001; Tchouankui, 2014; Deya-yang, 2016; Kombou, 2016; Metou, 2016; Kouam et al., 2018b; Djiojo, 2019); [e]: (Nghonjuyi et al., 2014; Bagari, 2019; Fokam, 2019).

Table S13. Characteristics and distribution of studies reporting coccidiosis in animals in Cameroon

Characteristics	Chicken	Small ruminants	Pigs	Cattle	Total
Number of studies	8[a]	4[b]	5[c]	3[d]	18
Region					
Adamawa	1	/	1	/	2
Centre	/	1	/	/	1
Far North	/	/	1	/	1
Littoral	/	1	/	/	1
North	/	/	/	1	1
North West	/	/	/	1	1
South West	1	/	/	/	1
West	6	2	3	1	10
Sample	Faeces				
Diagnosis	Coprology				

Legend: [a]: (Nghonjuyi et al., 2014; Dongmo, 2017b; Mbiakop, 2018; Mbitkebeyo, 2018; Bagari, 2019; Fokam, 2019; Mbotake, 2019; Ngadjie, 2019); [b]: (Simeni, 2014; Tchouagoue, 2014; Tchouankui, 2014; Mbiakop, 2018); [c]: (Deyayang, 2016; Kombou, 2016; Metou, 2016; Kouam et al., 2018b; Djiojo, 2019); [d]: (Dicko, 2015; Mamoudou et al., 2015c; Mbotake, 2019).

Table S14. Species of ticks reported in Cameroon

Characteristics		Cattle	Small ruminants	Total
Number of studies		7[a]	1[b]	8
Region				
Adamawa		6	/	6
North West		1		1
South		/	/	1
Species identified				
Amblyomma variegatum		4	1	5
Rhipicephalus (Boophilus) spp	*Rh. microplus,*	2	/	2
	Rh. Sanguineus	1	1	2
	Rh. Evertsi	/	1	1
	Rh. geigyi,	3	/	3
	Rh. Annulatus	3	/	3
	Rh. Decoloratus	3	/	3
	Rh. Appendiculatus	1	/	1
	Not identified	2	/	2
Hyalomma spp	*H. rufpes,*	2	/	2
	H. truncatum	2	/	2
	Not identified	2	/	2
Haemophysalis laechi		1	/	1

Legend: [a]: (Achukwi et al., 2001; Awa et al., 2015; Mamoudou et al., 2015b; Mamoudou et al., 2016a; Mamoudou et al., 2016b; Manchang et al., 2016; Silatsa et al., 2019) ; [b]: (Kouam and Dongmo, 2018).

Table S15. Characteristics and distribution of studies reporting brucellosis in animals in Cameroon

Species	Region	Number of studies (references)	Sample	Diagnostic technique	Risk factors
Cattle[a]	Adamawa	6	Blood: 5<br>Milk: 1	RBPT: 1<br>ELISA: 5	Presence of other species (1), herd size (1), locality (1), history of third trimester abortion (1), Husbandry system (2)
	North West	6	Blood: 5<br>Milk: 1	RBPT: 2<br>ELISA: 4	
	West	2	Blood	RBPT	
	East	1		RBPT	
Small ruminants[b]	Adamawa	1	Blood	ELISA	
Pigs[c]	Far North	1	Blood	ELISA	
	Littoral	1	Blood	ELISA	
Total	7	12	Blood:11<br>Milk: 1	RBPT: 2<br>ELISA: 12	

Legend : [a]: (Bayemi P. H. et al., 2009; Scolamacchia et al., 2010; Ojong, 2011; Bayemi et al., 2015; Kong et al., 2016; Ojong et al., 2016a; Awah-Ndukum et al., 2018a; Awah-Ndukum et al., 2018b; Loyem, 2019; Musallam et al., 2019); [b]: (Saya, 2017); [c]: (Moiffo, 2016).

Table S16. Regional pooled prevalence of brucellosis in cattle in Cameroon

0Region	Number of study reports included in meta-analysis	Pooled prevalence (95%CI)	P-value
North West	3	5.8 (4.0-8.5)	0.39
Adamawa	4	8.4 (3.6-18.4)	

Table S17. Characteristics and distribution of studies reporting tuberculosis in animals in Cameroon

	Region	Number of studies (references)	Sample	Diagnostic technique
Cattle[a]	Adamawa	2	Carcass	PM examination
	Far North	1	Carcass	PM examination
	North	1	Carcass	PM examination
	Littoral	4	Carcass	PM examination
	Centre	2	Carcass	PM examination
	West	2	Carcass	PM examination
	North West	4	Skin (intradermal injection): 2	Tuberculin skin test: 2
			Carcass: 2	PM examination: 2
Goats[b]	North	1	Skin(intradermal injection)	Tuberculin skin test
	Total	11	Carcass: 8	PM examination: 8

		Skin (intradermal injection): 3	Tuberculin skin test: 3

Legend: [a]: (Awah-Ndukum et al., 2005; Awah-Ndukum et al., 2010b; Awah-Ndukum et al., 2012a; Awah-Ndukum et al., 2012b; Awah-Ndukum et al., 2012c; Francioli et al., 2013; Egbe et al., 2016; Ngoudjou, 2018; Yemelong, 2019; Francioli et al., 2013; Kouengoua, 2014; Egbe et al., 2016; Manchang et al., 2016); [b]: (Tchedele, 2017).

Table S18. Pooled regional prevalence of bovine tuberculosis

Region	Number of studies	Pooled prevalence (95%CI)	P-value
North West	5	2.9 (1.7-4.7)	0.66
West	2	3.6 (2.3-5.5)	
Centre	2	1.7 (0.4-7.1)	
Littoral	3	2.9 (0.3-25.1)	

Table S19. Characteristics and distribution of studies reporting salmonellosis, colibacillosis and campylobacteriosis in animals in Cameroon

Infection	Characteristics	Chicken	Cattle	Pigs	Total
Salmonellosis	Number of studies	3[a]	2[b]	2[c]	6
	Region				
	Adamawa	1	/	/	1
	Far North	/	1	/	1
	West	1	/	1	1
	South West	/	1	1	1
	Centre	1	/	/	1
	Samples	Faeces			
	Diagnostic techniques	Bacterial culture			
Colibacillosis	Number of studies	2d	/	/	2
	Regions				
	Centre	1	/	/	
	Adamawa	1	/	/	
	Samples	Faeces			
	Diagnostic techniques	bacterial culture			
Campylobacteriosis	Number of studies	2e			2
	Region				
	Centre	1			
	Samples	Faeces, Neck skin	/	/	
	Diagnostic techniques	Bacterial culture		/	
Risk factors		presence of rodents (1), farm hygiene (1), water supplies and methods of feed production (1), farm location (1)			

Legend : [a]: (Nzouankeu et al., 2010; Bouba, 2014; Kouam et al., 2019a) ; [b]: (Akoachere et al., 2009; Kochakbe, 2016); [c]: (Akoachere et al., 2009; Djuidje, 2019b); [d]: (Nzouankeu et al., 2010; Bouba, 2014); [e]: (Nzouankeu et al., 2010).

Table S20. Characteristics and distribution of studies reporting other bacterial diseases in animals in Cameroon

Disease	Host species	Region	Studies included	Sample	Diagnostic techniques	Species identified
Leptospirosis	Cattle	AD	2a	Serum	ELISA	
Pasteurellosis	Cattle	AD, FN, NW	1b	Serum	hemagglutination tests	serotypes (A5, A7, B1, B6 et E6).
CCPP	Goats	N	1c	Blood	ELISA	
CBPP	Cattle	N	1d	Blood	PCR	
Anthrax	Cattle	N, E, FN	1e	Blood	PCR	
Q Fever	Cattle	AD	1f	Banked serum	ELISA	
Mastitis	Cattle	AD	1g	Milk	Bacterial culture	*
Endometritis	Cattle	AD	1h	Uterus	Visual examination	**

Legend: [a]: (Scolamacchia et al., 2010; Ngu-Ngwa et al., 2020); [b]: (Njongmeta et al., 2001); [c]: (Haman, 2019); [d]: (Talba, 2014); [e]: (Pilo et al., 2011); [f]: (Scolamacchia et al., 2010); [g]: (Lewong, 2016); [h]: (Njanja, 2018); * Staphylococcus aureus, Staphylococcus on Negative Coagulase *Escherichia coli, Enterococcus faecalis*; ** *Citrobacter braakii, Actinomyces pyogenes, Proteusmirabilis, Enterobacter cloacae, Escherichia coli, Aeromonasn hydrophila, Bulkhoderia cepacia, Provendencia stuartii* and *Salmonella*.

Table S21. Characteristics and distributions of studies reporting FMD in animals in Cameroon

	Cattle	Sheep	Goats	Pigs	Total
Number of studies	9[a]	3[b]	2[c]	2[c]	13
Region					
Adamawa	6	2	1	1	6
East	1	/	/	/	1
Far North	1	/	/	1	2
North	1	1	1	/	2
Samples					
Blood	4	2	1	1	6
Live animals	3	/	/	/	3
Epithelial swabs	2	1	1	1	2
Probing swabs	2	1	/	1	2
Diagnostic techniques					
ELISA	4	2	1	1	6
PCR	3	/	/	/	3
Visual examination	5*	1	1	1	5
Risk factors					
Breed	2	/	/	/	2
Breeding system	2	/	/	/	2
vaccination history	1	/	/	/	1
Body condition	1	/	1	/	1
Presence of other animal species	1	/	/	/	1

Locality	/	/	1	1	2
Species	/	/	1	/	1

Legend: *in two of these studies, diagnosis was based on herdsmen reports; [a]: (Bronsvoort et al., 2003; Bronsvoort et al., 2004; Bronsvoort et al., 2006; Kenton et al., 2014; Ludi et al., 2014; Modjo, 2014; Kuate, 2016; Manchang et al., 2016; Beloko, 2019; Sevidzem et al., 2019); [b]: (Bronsvoort et al., 2004; Nsangou, 2017; Sevidzem et al., 2019); [c]: (Bronsvoort et al., 2004; Nsangou, 2017); [d]: (Bronsvoort et al., 2004; Dongmo, 2016).

Table S22. Characteristics and distribution of studies reporting SRP in small ruminants in Cameroon

	Sheep	Goats	Total
	3[a]	2[b]	3
Region			
Adamawa	1	/	1
North	1	/	1
West	1	1	1
Whole territory	1	1	1
Sample	Blood		
Diagnostic technique	ELISA		
Risk factors	mode of acquisition (1), vaccination status (1) sex (1), age(1), physiological state (1), agro-ecological zone (1), farming method (1) and field grazing (1)		

Legend : [a]: (Yongue, 2014; MINEPIA et al., 2016; Manglang, 2019) ; [b]: (Yongue, 2014; MINEPIA et al., 2016).

Table S23. Characteristics and distribution of studies reporting RVF in Cameroon

Study	Region		Samples	Diagnostic techniques	Risk factors
	Cattle	Small ruminants			
(Bagninbom, 2016)	NW, AD	/	Blood	ELISA	Interaction with other herds and wildlife species, the altitude of cattle rearing site, age, transhumance, grazing in flooded areas
(Poueme et al., 2019)	/	N	Blood	ELISA	Locality, age, access to water
(Rissmann et al., 2017)	N, FN, AD, C, S	N, FN, C, L, SW, W, NW, S	Blood	ELISA	/

Table S24. Characteristics and distribution of studies reporting ASF and other viral diseases of pigs in Cameroon

Disease	Region	Sample	Diagnostic techniques	Risk factors	References
ASF	AD, N, FN	Blood, tissues	PCR, ELISA		(Nguena, 2017)
	W	Blood	PCR	lack of footbath at the entrance, propagation by the farmer or veterinarian	(Nkana, 2014)
	S, C, SW	Blood, tissues	PCR, ELISA	/	(Abdelrazak, 2019)
	Whole territory	Blood, tissues, sera	PCR, ELISA	/	(Wade, 2019)
Influenza A virus H1N1	C, W, N, FN	Nasal swabs and sera	PCR	Free-roaming	(Larison et al., 2014)
	C, N	Nasal swabs	PCR		(Njabo et al., 2010)
Hepatitis E virus	N, W	Blood	ELISA	/	(Modiyinji et al., 2018)
	NW, C, L	Liver samples	PCR	/	(De Paula et al., 2013)
Porcine hokovirus	NW, L, C	Liver samples	PCR	/	(Adlhoch et al., 2013)
Novel Porcine bocaviruses	Not precise	Faeces	PCR	/	(Ndze et al., 2013)

Table S25. Characteristics and distribution of studies reporting other viral diseases of cattle in Cameroon

	Study	Region	Sample	Diagnostic technique (s)	Risk factors
Bovine viral diarrhoea	1[a]	AD	Blood	ELISA and PCR	Proximity of herds to other animal species (goats, antelopes…) during grazing or otherwise
Infectious Bovine Rhinotracheitis (IBR)	1[b]	N	Blood	ELISA	Locality, sex and age
Lumpy skin disease	1[c]	AD	Skin	AM examination	/

Legend: [a]: (Handel et al., 2011); [b]: (Abdoulaye, 2018); [c]: (Manchang et al., 2016).

Table S26. Characteristics and distribution of studies reporting viral diseases of poultry in Cameroon

Disease	Region	Sample	Diagnostic technique	Aetiological findings	Risk factors	References
Avian influenza H5N1	Whole territory	carcasses, swabs, eggs, tissues and droppings	avian flu rapid test	Epizooties fall within the genetic clade 2.3.2.1c	/	(Wade et al., 2018)
	West , Centre	/	/	/	origin of feed, absence of sanitary care, origin of chicks, absence of foot bath and inadequate transport means	(Zangho, 2017)
Avian influenza H5N8	Far North	/	/			(Wade, 2017)
Newcastle's disease	NW	Faeces, tracheal and cloacal swabs	PCR	genotypes XIV, XVII and XVIII	/	(Snoeck et al., 2013)
Chicken anaemia virus	Not precised		Serology	/	/	(Snoeck et al., 2012)

Supplementary S1. Figures

Parasitic diseases situation of livestock in Cameroon

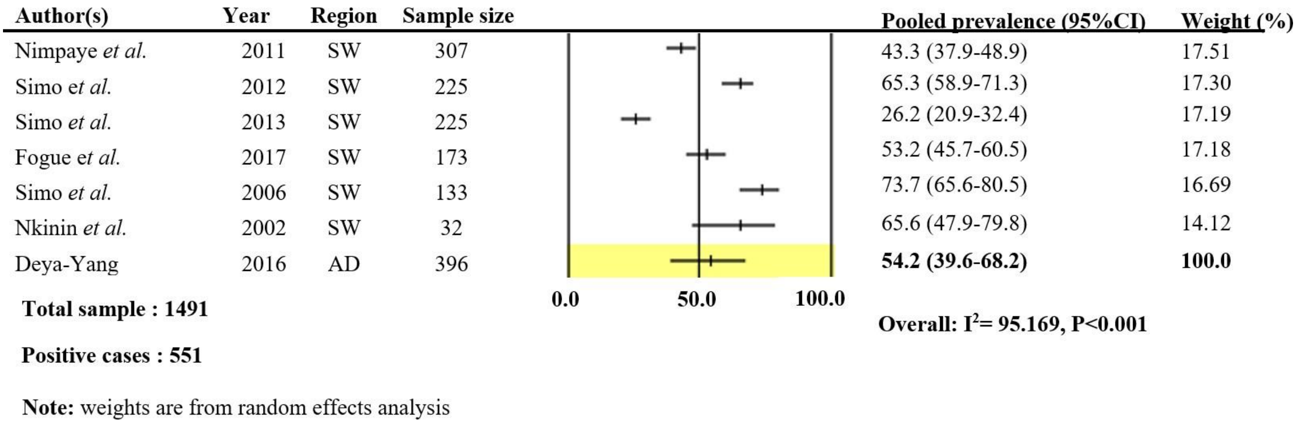

Figure S1. Forest plot of pooled prevalence of trypanosomiasis in pigs

Author(s)	Year	Region	Sample size	Pooled prevalence (95%CI)	Weight (%)
Nimpaye *et al.*	2011	SW	264	11.6 (8.3-16.0)	20.57
Simo e*t al.*	2012	SW	87	66.2 (53.9-76.6)	20.06
Simo *et al.*	2013	SW	87	10.8 (5.2-20.9)	18.74
Fogue e*t al.*	2017	SW	50	27.6 (17.6-40.4)	19.79
Diezoumbe	2017	AD	475	43.6 (36.9-50.5)	20.84
				**28.4 (12.7-51.9)**	**100.0**

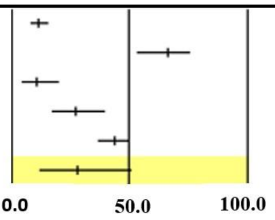

Total sample : 963

Positive cases : 270

Overall: I²= 95.934, P<0.001

Note: weights are from random effects analysis

Figure S2. Forest plot of pooled prevalence of trypanosomiasis in small ruminants

Author(s)	Year	Region	Sample size	Pooled prevalence (95% CI)	Weight (%)
Mamoudou *et al.*	2016d	AD	355	78.3 (73.7-82.3)	16.71
Mamoudou *et al.*	2015c	N	223	33.6 (27.7-40.1)	16.55
Ntonifor *et al.*	2013	NW	277	56.7 (50.8-62.4)	16.79
Mamoudou *et al.*	2018	AD	175	64.6 (57.2-71.3)	16.34
Dicko	2014	NW	200	50.0 (43.1-56.9)	16.55
Mbotake	2019	W	460	57.8 (53.3-62.3)	17.06
				**57.4 (45.3-68.7)**	**100.0**

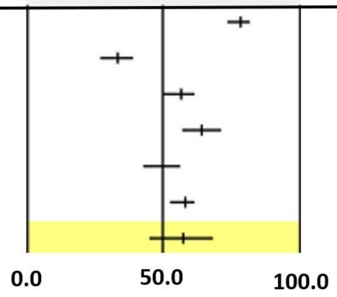

Total sample : 1690

Positive cases : 989

Overall: I²= 95.622, P<0.001

Note: weights are from random effects analysis

Figure S3. Forest plot of pooled prevalence of gastro intestinal parasites in cattle in Cameroon

Author(s)	Year	Region	Sample size	Pooled prevalence (95% CI)	Weight (%)
Kelly et al.	2019	AD	1112	56.4 (53.5-59.3)	6.44
Kelly et al.	2019	NW	776	0.6 (0.3-1.5)	5.47
Takang et al.	2019	C	767	18.0 (15.4-20.9)	6.41
Kouam et al.	2018a	SW	1472	22.6 (20.6-24.8)	6.44
Chahdini et al.	2020	AD	1361	13.2 (11.5-15.1)	6.43
Manchang et al.	2016	AD	4072	26.9 (25.5-28.2)	6.46
Fotso	2014	L	4731	5.1 (4.5-5.7)	6.44
Mbotake	2019	W	460	15.7 (12.6-19.3)	6.37
Dicko	2014	NW	200	6.5 (3.8-10.9)	6.02
Kelly et al.	2018	AD	732	49.6 (46.0-53.2)	6.43
Ekoual	2019	AD	80	46.3 (35.7-57.2)	6.18
Tebug	2009	NW	1449	2.8 (2.0-3.7)	6.32
Yemelong	2019	W	382	2.64 (2.23-3.11)	6.39
Nzale	2014	W	241	2.1 (0.9-4.9)	5.46
Salhine	2016	AD	175	66.9 (59.6-73.4)	6.32
Abakar	2014	AD	500	43.6 (39.3-48.0)	6.42
				**17.3 (11.0-26.2)**	**100.0**

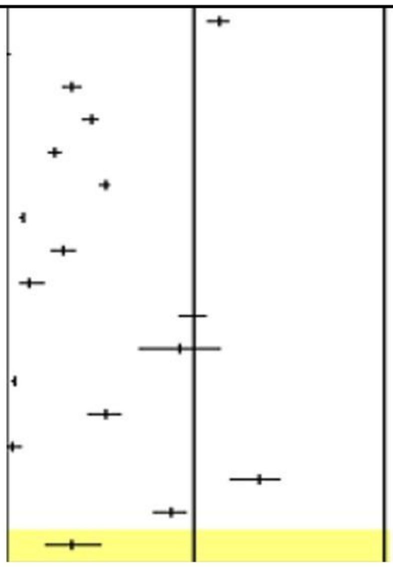

Total sample: 18510

Positive cases: 3581

Overall: I²: 99.39, p<0.001

Note: weights are from random effects analysis

Figure S4. Forest plot of pooled prevalence of fascioliasis in cattle in Cameroon

Author(s)	Year	Region	Sample size	Pooled prevalence (95% CI)	Weight (%)
Allo *et al.*	2012	NW	499	7.6 (5.6-10.3)	11.55
Pouedet *et al.*	2002	W	707	11.0 (8.9-13.6)	11.73
Assana *et al.*	2010	N	398	24.6 (20.6-29.1)	11.74
Assana *et al.*	2019a	C	416	8.7 (6.3-11.8)	11.53
Assana *et al.*	2001	N	139	38.8 (31.1-47.2)	11.53
Dongmo	2019	W	502	0.1 (0.0-0.16)	3.80
Azebaze	2000	W	251	3.2 (1.6-6.2)	10.47
Assana *et al.*	2019b	C	305	8.9 (6.1-12.6)	11.41
Tchinda	2000	W	264	3.0 (1.5-5.9)	10.47
Manchang	2016	N	4072	0.0 (0.0-0.2)	5.77
**Total sample : 7553**				**6.2 (3.3-11.3)**	**100.0**
**Positive cases : 348**				**Overall: I^2= 96.092, P<0.001**	

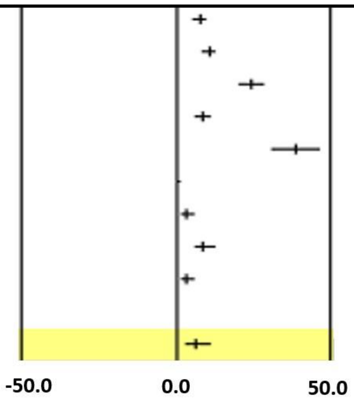

Note: weights are from random effects analysis

Figure S5. Forest plot of pooled prevalence of porcine cysticercosis in Cameroon

Bacterial diseases situation of livestock in Cameroon

Author(s)	Year	Region	Sample size	Pooled prevalence (95% CI)	Weight (%)
Scolamacchia et al.	2010	AD	1377	3.1 (2.3-4.2)	10.28
Awah-Ndukum et al.	2018b	AD	590	5.9 (4.3-8.2)	10.11
Awah-Ndukum et al.	2018a	AD	522	11.5 (9.0-14.5)	10.41
Awah-Ndukum et al.	2018a	N	509	6.1 (4.3-8.5)	10.01
Bayemi et al.	2015	NW	689	5.2 (3.8-7.2)	10.14
Bayemi et al.	2009	NW	266	8.3 (5.5-12.2)	9.65
Kong et al.	2016	NW	198	4.0 (2.0-7.9)	8.10
Shey-Njila et al.	2005	C	840	9.6 (7.8-11.8)	10.55
Ojong et al.	2011	AD, E, NW, W	1562	4.6 (3.7-5.8)	10.53
Loyem	2019	AD	223	21.5 (16.6-27.4)	10.21
**Total sample: 6776**				**7.0 (4.8-10.0)**	**100.0**
**Positive cases: 436**				**Overall: I^2= 93.514, P<0.001**	

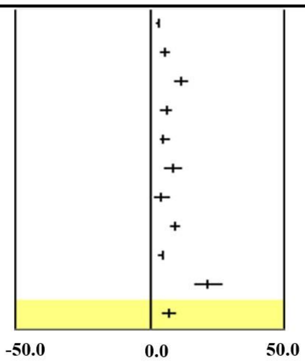

Note: weights are from random effects analysis

Figure S6. Forest plot of pooled prevalence of brucellosis in cattle in Cameroon

Author(s)	Year	Region	Sample size	Pooled prevalence (95% CI)	Weight (%)
Poueme et al.	2019	N	355	7.1 (1.0-37.0)	15.37
Poueme et al.	2019	N	325	2.4 (0.3-15.1)	28.12
Rissmann et al.	2017	FN	14	4.9 (2.8-8.7)	1.82
Rissmann et al.	2017	N	42	1.2 (0.2-8.1)	1.92
Rissmann et al.	2017	C	223	6.7 (2.2-18.7)	20.55
Rissmann et al.	2017	L	82	1.9 (0.3-12.0)	1.94
Rissmann et al.	2017	SW	45	2.9 (1.3-6.2)	5.50
Rissmann et al.	2017	W	54	3.4 (1.5-7.4)	1.93
Rissmann et al.	2017	NW	210	2.3 (1.1-4.4)	11.46
Rissmann et al.	2017	S	175	4.6 (2.8-7.5)	11.39
**Total sample: 1525**				**3.7 (2.9-4.9)**	**100.0**

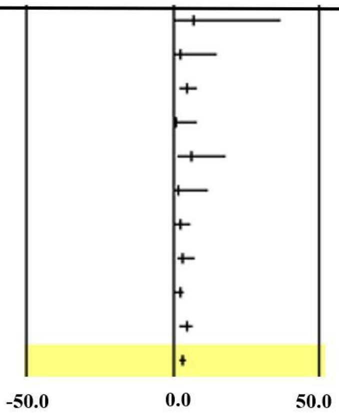

Positive cases: 53

Overall: I^2= 0.000, P=0.566

Note: weights are from random effects analysis

Figure S7. Forest plot of pooled prevalence of RVF in small ruminants in Cameroon

Author(s)	Year	Region	Pooled prevalence (95% CI)	Weight (%)
Rissmann et al.	2017	FN	19.1 (14.7-24.5)	17.66
Rissmann et al.	2017	N	11.8 (9.1-15.0)	18.16
Rissmann et al.	2017	AD	11.7 (8.3-16.2)	16.64
Rissmann et al.	2017	C	9.1 (1.3-43.9)	2.47
Rissmann et al.	2017	S	10.9 (4.6-23.6)	8.26
Bagninbom	2016	NW	6.9 (5.3-9.0)	18.20
Bagninbom	2016	AD	8.7 (6.9-10.9)	18.62
			**10.9 (8.0-14.7)**	**100.0**

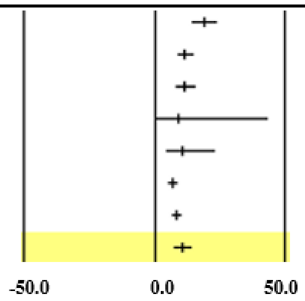

Total sample: 2532

Positive cases: 256

Overall: I^2= 81.722, P<0.001

Note: weights are from random effects analysis

CI: Confidence interval

Figure S8. Forest plot of pooled prevalence of RVF in cattle in Cameroon

Author(s)	Year	Region	Sample size	Pooled prevalence (95% CI)	Weight (%)
Nguena	2017	AD, N, FN	688	5.2 (3.8-7.2)	16.98
Nkana	2014	W	100	7.0 (3.4-14.0)	15.32
Abdelrazak	2019	C	441	27.9 (23.9-32.3)	17.26
Abdelrazak	2019	S	185	11.9 (8.0-17.4)	16.66
Abdelrazak	2019	SW	58	46.6 (34.2-59.3)	16.41
Wade et al.	2019	whole territory	1000	42.8 (39.8(45.9)	17.37
				**18.9 (8.9-35.6)**	**100.0**

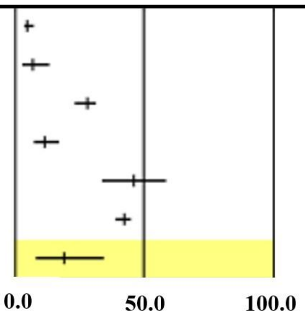

Total sample: 2472

Positive cases: 643

Overall: $I^{2=}$ 98.15; p<0.001

Note: weights are from random effects analysis

Figure S9. Forest plot of pooled prevalence of African swine fever in pigs in Cameroon

Supplementary S2. Prisma check list used

Section and Topic	Item #	Checklist item	Location where item is reported
**TITLE** **Zoo-sanitary situation assessment, an initial step in country disease prioritization process: Systematic review and meta-analysis from 2000 to 2020 in Cameroon**			
Title	1	Identify the report as a systematic review.	done
**ABSTRACT** To prevent and/or control infectious diseases in animal and human health, an appropriate surveillance system based on suitable up-to-date epidemiological data is required. The systematic review protocol was designed according to the PRISMA statement to looks at the available data on infectious diseases of livestock in Cameroon from 2000-2020. Data was searched through online databases. Grey literature comprised of dissertations and thesis from veterinary higher education institutions in Cameroon. A random-effects model was used to calculate pooled prevalence using Comprehensive Meta-analysis software. Based on disease prevalence, major infectious diseases of livestock in Cameroon were gastrointestinal parasitosis (57.4% in cattle, 67.2% in poultry, 88% in pigs), hemoparasites (21.6% in small ruminants, 19.7% in cattle), bovine pasteurellosis (55.5%), fowl salmonellosis (48.2%), small ruminant plague (39.7%), foot-and-mouth disease (34.5% in cattle) and African swine fever (18.9%). Furthermore, other important endemic zoonoses in the country included: Rift Valley fever (10.9% in cattle, 3.7% in small ruminants), brucellosis (7% in cattle, 8% in pigs), bovine tuberculosis (4.7% in cattle), hepatitis E virus (8.4% in pigs) and bovine leptospirosis (2.5%). Most of the retrieved research were carried out in the Adamawa, North-West and West regions of Cameroon. The evaluation of existing data as evidence, albeit publication-specific, is an important step towards the process of prioritizing animal diseases, including zoonoses.			
Abstract	2	See the PRISMA 2020 for Abstracts checklist.	Respect of the prisma checklist
**INTRODUCTION**			
Rationale	3	Describe the rationale for the review in the context of existing knowledge.	Done
Objectives	4	Provide an explicit statement of the objective(s) or question(s) the review addresses.	Done

**METHODS**			
Eligibility criteria	5	Specify the inclusion and exclusion criteria for the review and how studies were grouped for the syntheses.	Done
Information sources	6	Specify all databases, registers, websites, organisations, reference lists and other sources searched or consulted to identify studies. Specify the date when each source was last searched or consulted.	Done
Search strategy	7	Present the full search strategies for all databases, registers and websites, including any filters and limits used.	Done
Selection process	8	Specify the methods used to decide whether a study met the inclusion criteria of the review, including how many reviewers screened each record and each report retrieved, whether they worked independently, and if applicable, details of automation tools used in the process.	Done
Data collection process	9	Specify the methods used to collect data from reports, including how many reviewers collected data from each report, whether they worked independently, any processes for obtaining or confirming data from study investigators, and if applicable, details of automation tools used in the process.	Done
Data items	10a	List and define all outcomes for which data were sought. Specify whether all results that were compatible with each outcome domain in each study were sought (e.g. for all measures, time points, analyses), and if not, the methods used to decide which results to collect.	Done
	10b	List and define all other variables for which data were sought (e.g. participant and intervention characteristics, funding sources). Describe any assumptions made about any missing or unclear information.	Done
Study risk of bias assessment	11	Specify the methods used to assess risk of bias in the included studies, including details of the tool(s) used, how many reviewers assessed each study and whether they worked independently, and if applicable, details of automation tools used in the process.	Done
Effect measures	12	Specify for each outcome the effect measure(s) (e.g. risk ratio, mean difference) used in the synthesis or presentation of results.	Done
Synthesis methods	13a	Describe the processes used to decide which studies were eligible for each synthesis (e.g. tabulating the study intervention characteristics and comparing against the planned groups for each synthesis (item #5)).	Done
	13b	Describe any methods required to prepare the data for presentation or synthesis, such as handling of missing summary statistics, or data conversions.	Done
	13c	Describe any methods used to tabulate or visually display results of individual studies and syntheses.	Done
	13d	Describe any methods used to synthesize results and provide a rationale for the choice(s). If meta-analysis was performed, describe the model(s), method(s) to identify the presence and extent of statistical heterogeneity, and software package(s) used.	Done
	13e	Describe any methods used to explore possible causes of heterogeneity among study results (e.g. subgroup analysis, meta-regression).	Done
	13f	Describe any sensitivity analyses conducted to assess robustness of the synthesized results.	Done
Reporting bias assessment	14	Describe any methods used to assess risk of bias due to missing results in a synthesis (arising from reporting biases).	Done
Certainty assessment	15	Describe any methods used to assess certainty (or confidence) in the body of evidence for an outcome.	Done

RESULTS			
Study selection	16a	Describe the results of the search and selection process, from the number of records identified in the search to the number of studies included in the review, ideally using a flow diagram.	Done
	16b	Cite studies that might appear to meet the inclusion criteria, but which were excluded, and explain why they were excluded.	Done
Study characteristics	17	Cite each included study and present its characteristics.	Done
Risk of bias in studies	18	Present assessments of risk of bias for each included study.	Done
Results of individual studies	19	For all outcomes, present, for each study: (a) summary statistics for each group (where appropriate) and (b) an effect estimate and its precision (e.g. confidence/credible interval), ideally using structured tables or plots.	Done
Results of syntheses	20a	For each synthesis, briefly summarise the characteristics and risk of bias among contributing studies.	Done
	20b	Present results of all statistical syntheses conducted. If meta-analysis was done, present for each the summary estimate and its precision (e.g. confidence/credible interval) and measures of statistical heterogeneity. If comparing groups, describe the direction of the effect.	Done
	20c	Present results of all investigations of possible causes of heterogeneity among study results.	Done
	20d	Present results of all sensitivity analyses conducted to assess the robustness of the synthesized results.	Done
Reporting biases	21	Present assessments of risk of bias due to missing results (arising from reporting biases) for each synthesis assessed.	Done
Certainty of evidence	22	Present assessments of certainty (or confidence) in the body of evidence for each outcome assessed.	Done

DISCUSSION			
Discussion	23a	Provide a general interpretation of the results in the context of other evidence.	Done
	23b	Discuss any limitations of the evidence included in the review.	Done
	23c	Discuss any limitations of the review processes used.	Done
	23d	Discuss implications of the results for practice, policy, and future research.	Done
**OTHER INFORMATION**			-
Registration and protocol	24a	Provide registration information for the review, including register name and registration number, or state that the review was not registered.	Not registered
	24b	Indicate where the review protocol can be accessed, or state that a protocol was not prepared.	
	24c	Describe and explain any amendments to information provided at registration or in the protocol.	
Support	25	Describe sources of financial or non-financial support for the review, and the role of the funders or sponsors in the review.	IMMANA
Competing interests	26	Declare any competing interests of review authors.	No competing interest
Availability of data, code and other materials	27	Report which of the following are publicly available and where they can be found: template data collection forms; data extracted from included studies; data used for all analyses; analytic code; any other materials used in the review.	Not define

Supplementary S3. Study quality assessment form and decision

Question 1: was the basic data, including study period, sample type, disease condition, and study site, provided?

Question 2: Were the study participants recruited appropriately?

Question 3: Was the sample size representative of the target population (for cross-sectional studies alone)?

Question 4: Were the study subjects and setting described in detail?

Question 5: Was the data analysis conducted with sufficient coverage of the identified pathogen?

Question 6: Were all important confounding factors/subgroups/differences identified and accounted for?

Question 7: Were objectives and standard criteria used to measure the condition?

Question 8: Was the condition measured reliably (with valid methods and trained personnel)?

9 Response coe: **Y**=yes; **N**=no", **I**=irrelevant; **U**=unclear 11

**N°**	**Study**	**Question number**								**Decision to include**
		**1**	**2**	**3**	**4**	**5**	**6**	**7**	**8**	
1.	Abakar, A. A. (2014). *Prévalence et impacts économiques de la fasciolose à l'abattoir municipal de Ngaoundéré [Unpublished doctoral dissertation].* (Doctor of Veterinary Medicine), School of Veterinary Medicine and Sciences, University of Ngaoundere, Cameroon.	Y	Y	U	U	Y	Y	Y	Y	High
2.	Abanda, B., Paguem A., Abdoulmoumini M., Manchang T. K., Renz A., & Eisenbarth A. (2019). Molecular identifcation and prevalence of tick-borne pathogens in zebu and taurine cattle in North Cameroon. *Parasites & Vectors, 12*(448), https://doi.org/10.1186/s13071-13019-13699-x..	Y	Y	U	U	Y	Y	Y	Y	High
3.	Abdelrazak, A. (2019). *Etude épidémiologique de la Peste Porcine Africaine dans les régions du Centre, du Sud et du Sud-Ouest Cameroun, caractérisation moléculaire du virus et essais thérapeutiques [Unpublished doctoral dissertation].* (Doctor of Veterinary Medicine), School of Veterinary Medicine and Sciences, University of Ngaoundere, Cameroon.	Y	Y	Y	Y	Y	Y	Y	Y	High
4.	Abdoulaye, M. R. (2018). *Séroprévalence et facteurs de risque de la Rhinotrachéite Infectieuse Bovine dans le Département de la Bénoué (Région du Nord, Cameroun [Unpublished doctoral dissertation]).* (Doctor in Veterinary Medicine), School of Veterinary medicine and Sciences, University of Ngaoundere, Cameroon.	Y	Y	Y	Y	N	Y	Y	Y	High

5.	Achukwi, M. D., & Musongong, G. A. (2009). Trypanosomosis in the Doayo/Namchi (Bos taurus) and zebu White Fulani (Bos indicus) cattle in Faro Division, North Cameroon. *Journal of Applied Biosciences 15*, 807-814.	Y	N	I	Y	Y	Y	I	Y	High
6.	Adlhoch, C., Kaiser M., Manchang T. K., Schwarz N. G., Ulrich M., de Paula V. S., & Ellerbrok H. (2013). Porcine Hokovirus in Domestic Pigs, Cameroon. *Emerging Infectious Diseases*, *19*(12), 2060-2062 http://dx.doi.org/2010.3201/eid1912.130891.	Y	N	I	U	Y	Y	I	Y	High
7.	Akoachere, J.-F., Tanih N. F., Ndip L. M., & N., N. R. (2009). Phenotypic Characterization of Salmonella Typhimurium Isolates from Food-animals and Abattoir Drains in Buea, Cameroon. *Journal of Health, Population and Nutrition, 27* (5), 612-618.	Y	U	I	U	N	Y	I	Y	High
8.	Aliyou, H. (2014). *Etude épidémiologique de la trypanosomose bovine dans le mayo-rey région du nord cameroun [Unpublished doctoral dissertation]*. (Doctor of Veterinary Medicine), School of Veterinary Medicine and Sciences, University of Ngaoundere, Cameroon.	Y	Y	Y	Y	Y	Y	Y	Y	High
9.	An’naguil, T. I. (2017). *Identification, prévalence et facteurs de risques des hémoparasites transmis par les tiques aux petits ruminants dans le Département de la Bénoué (Région du Nord Cameroun [Unpublished doctoral dissertation]).* (Doctor of Veterinary Medicine), School of Veterinary Medicine and Sciences, University of Ngaoundere, Cameroon.	Y	Y	Y	Y	Y	Y	Y	Y	High
10.	Anoumbo, A. M. A. (2017). *Prévalence et intensité d'infestation des helminthes gastro-intestinaux et hémoparasites des petits ruminants en fonction des types d'élevages dans le départment de la Vina [Unpublished doctoral dissertation].* (Doctor of Veterinary Medicine), School of Veterinary medicine and Sciences, Cameroon.	Y	Y	U	U	Y	Y	Y	Y	High
11.	Assana, E., Amadou F., Thys E., Lightowlers M. W., Zoli A. P., Dorny P., & Geerts S. (2010). Pig-farming systems and porcine cysticercosis in the north of Cameroon. *Journal of helminthology* (84), 441–446.	Y	Y	Y	Y	Y	Y	N	Y	High
12.	Assana, E., Awah-Ndukum J., Djonmaïla D. J., & Zoli P. A. (2019). Prevalence of porcine Taenia solium and Taenia hydatigena cysticercosis in Cameroon. *Preventive Veterinary Medicine*,169, 104690.	Y	Y	Y	U	Y	Y	N	Y	High
13.	Assana, E., Awah-Ndukum J., Djonmaila J. D., Djiatche H. D., Awé C., Manchang T. K., & Zoli P. A. (2019). A comparison of Taenia solium and Taenia hydatigena infection in pigs using serological diagnosis and post-mortem inspection methods in Benoué division, North Cameroon. *Veterinary Parasitology: Regional Studies and Reports*, 17, 100306, https://doi.org/10.1016/j.vprsr.2019.100306.	Y	Y	Y	Y	N	Y	U	Y	High
14.	Assana, E., Zoli P. A., Sadou H. A., Nguekam, Vondou L., Pouedet M. S. R., Geerts S. (2001). Prévalence de la cysticercose porcine dans le Mayo-Danay (Nord Cameroun) et le Mayo-Kebbi (sud-ouest du Tchad). *Revue d'élevage et de médecine vétérinaire des pays tropicaux, 54*(2), 123-127.	Y	Y	Y	U	Y	Y	N	Y	High
15.	Awa, D. N., & Ndamkou, C. N. (2006). Response of Trypanosoma vivax and Trypanosoma congolense in zebu cattle in North Cameroon to prophylactic treatment with two formulations of isometamidium. *Preventive Veterinary Medicine, 76*, 90-96.	Y	Y	Y	Y	Y	Y	Y	Y	High
16.	Awa, D. N., Adakal H., Luogbou N. D. D., Wachong K. H., Leinyuy I., & Achukwi M. D. (2015). Cattle ticks in Cameroon: Is Rhipicephalus (Boophilus) microplus absent in Cameroon and the Central African region? *Ticks and Tick-borne Diseases, 6*, 117-122 http://dx.doi.org/110.1016/j.ttbdis.2014.1010.1005.	Y	I	I	Y	Y	Y	I	Y	High
17.	Awa, D. N., Ngagnou A., Tefiang E., Yaya D., & Njoya A. (2002). Post-vaccination and colostral peste des petits ruminants antibody dynamics in research flocks of kirdi goats and foiulbe sheep of North Cameroon. *Preventive Veterinary Medicine, 55*, 265-271	Y	N	U	N	Y	N	Y	Y	Medium

18.	Awah-Ndukum, J., Kudi A. C., Bah G. S., Bradley G., Tebug S. F., Dickmu P. L., & Agharih W. N. (2012). Bovine Tuberculosis in Cattle in the Highlands of Cameroon: Seroprevalence Estimates and Rates of Tuberculin Skin Test Reactors at Modified Cut-Offs. *Veterinary Medicine International*, doi:10.1155/2012/798502.	Y	Y	Y	Y	Y	Y	Y	Y	High
19.	Awah-Ndukum, J., Kudi A. C., Bradley G., Smith N. H., Ane-Anyangwe I., Cho-Ngwa F., & Titanji K. V. P. (2013). Molecular genotyping of Mycobacterium bovis isolated from cattle tissues in the North West Region of Cameroon. *Tropical Animal Health and Production*, 45, 829–836.	Y	Y	U	N	Y	Y	Y	Y	High
20.	Awah-Ndukum, J., Kudi A. C., Bradley G., Smith N. H., Ane-Anyangwe I., Cho-Ngwa F., & Titanji V. P. K. (2012). Molecular genotyping of Mycobacterium bovis isolated from cattle tissues in the North West Region of Cameroon. *Trop Anim Health Prod* (45), 829–836	Y	Y	Y	Y	Y	Y	Y	Y	High
21.	Awah-Ndukum, J., Kudi C., Bradley G., Ane-Anyangwe I. N., Fon-Tebug S., & J., T. (2010). Prevalence of Bovine Tuberculosis in Abattoirs of the Littoral and Western Highland Regions of Cameroon: A Cause for Public Health Concern. *Veterinary Medicine International, 2010*, doi:10.4061/2010/495015.	Y	Y	N	U	Y	Y	Y	Y	High
22.	Awah-Ndukum, J., Kudi C., Bradley G., Ane-Anyangwe I. N., Fon-Tebug S., & J., T. (2010). Prevalence of Bovine Tuberculosis in Abattoirs of the Littoral and Western Highland Regions of Cameroon: A Cause for Public Health Concern. *Veterinary Medicine International, 2010*, doi:10.4061/2010/495015.	Y	Y	Y	Y	N	Y	Y	Y	High
23.	Awah-Ndukum, J., Kudi, A. C., Bah, G. S., Bradley, G., Tebug, S. F., Dickmu, P. L., Njakoi, H. N., & Agharih, W. N. (2012). Bovine tuberculosis in cattle in the highlands of cameroon: seroprevalence estimates and rates of tuberculin skin test reactors at modified cut-offs. *Veterinary medicine international*, *2012*, 798502. https://doi.org/10.1155/2012/798502	Y	N	Y	Y	Y	Y	N	Y	High
24.	Awah-Ndukum, J., Mouiche M. M. M., Bayang H. N., Ngu Ngwa V., Assana E., Feussom K. J. M., & .Zoli P. A. (2018). Seroprevalence and Associated Risk Factors of Brucellosis among Indigenous Cattle in the Adamawa and North Regions of Cameroon. *Veterinary Medicine International, 2018*, https://doi.org/10.1155/2018/3468596.	Y	N	Y	Y	Y	Y	Y	Y	High
25.	Awah-Ndukum, J., Mouiche M. M. M., Kouonmo-Ngnoyum L., Bayang H. N., Manchang T. K., Poueme R. S. N., Zoli P. A. (2018). Seroprevalence and risk factors of brucellosis among slaughtered indigenous cattle, abattoir personnel and pregnant women in Ngaoundéré, Cameroon. *BMC Infectious Diseases 18*, 611.	Y	Y	Y	Y	Y	Y	Y	Y	High
26.	Awah-Ndukum, J., Tchoumboue J., & Niba A. T. (2005). Prevalence of bovine tuberculosis at the SODEPA Douala abattoir Cameroon (1995-2003). *Cameroon Journal of Experimental Biology*, *1*(02), 116-120.	Y	N	Y	Y	Y	Y	Y	Y	High
27.	Bagari, I. S. (2019). *Inventaire des helminthes digestifs et des coccidies chez les poules locales dans les lieux d'abattage de la ville de Ngaoundéré [Unpublished doctoral dissertation]*. (Doctor of Veterinary Medicine), School of Veterinary Medicine and Sciences, University of Ngaoundere, Cameroon.	Y	Y	U	Y	Y	N	Y	Y	High
28.	Bagninbom, J. M. (2016). *Séroprévalence et facteurs de risque de la fièvre de la vallée du Rift chez les bovins dans les hautes terres du Cameroun [Unpublished doctoral dissertation]*. (Doctor of Veterinary Medicine), School of Veterinary Medicine and Sciences, University of Ngaoundere, Cameroon.	Y	Y	Y	Y	Y	Y	Y	Y	High

29.	Bayang, H. N. (2014). *Prevalence et facteurs de risque de la brucellose bovine dans les régions du Nord et de l'Adamaoua, Cameroun [Unpublished doctoral dissertation].* (Doctor in Veterinary Medicine), School of Veterinary medicine and Sciences, University of Ngaoundere, Cameroon.	Y	Y	N	N	Y	Y	Y	Y	High
30.	Bayemi P. H., C., W. E., Nsongka M. V., Unger H., & Njakoi H. (2009). Prevalence of Brucella abortus antibodies in serum of Holstein cattle in Cameroon. *Tropical Animal Health and Production, 41*, 141–144.	Y	N	U	U	Y	Y	Y	Y	High
31.	Bayemi, P. H., Mah G. D., Ndamukong K., Songka V. M., Leinyuy I., Unger H., & Luogbou N. D. (2015). Bovine Brucellosis in Cattle Production Systems in the Western Highlands of Cameroon. *International Journal of Animal Biology, 1*(2), 38-44.	Y	Y	U	U	Y	Y	Y	Y	High
32.	Beloko, S. L. (2019). *Prévalence et facteurs de risques de la fièvre aphteuse bovine dans le marché à bétail de Garoua-Boulaï et dans la zone de transhumance du département du Lom et Djerem à l'Est-Cameroun [Unpublished doctoral dissertation].* (Doctor in Veterinary Medicine), School of Veterinary Medicine and Sciences, University of Ngaoundere, Cameroon.	Y	Y	N	N	Y	Y	Y	Y	High
33.	Bertram, M. R., Bravo de Rueda, C., Garabed, R., Dickmu Jumbo, S., Moritz, M., Pauszek, S., Abdoulkadiri, S., Rodriguez, L. L., & Arzt, J. (2018). Molecular Epidemiology of Foot-and-Mouth Disease Virus in the Context of Transboundary Animal Movement in the Far North Region of Cameroon. *Frontiers in veterinary science, 5*, 320. https://doi.org/10.3389/fvets.2018.00320	Y	Y	Y	N	N	Y	N	Y	Medium
34.	Bertram, M. R., Delgado A., Pauszek S. J., Smoliga G. R., Brito B., Stenfeldt C., & Arzt J. (2018). Effect of vaccination on cattle subclinically infected with foot-and-mouth disease virus in Cameroon. *Preventive Veterinary Medicine*, 155, 1-10. https://doi.org/10.1016/j.prevetmed.2018.1004.1003.	Y	Y	U	N	Y	Y	N	Y	High
35.	Bessong-Takang, N.-A. (2019). *Seroprevalence and Associated Risk Factors of Leptospirosis among Slaughtered Cattle and Personnel in the Ngaoundéré Municipal Abattoir [Unpublished doctoral dissertation].* (Doctor in Veterinary Medicine), School of Sciences and Veterinary Medicine, Cameroon.	Y	Y	Y	Y	Y	Y	Y	Y	High
36.	Bouba, M. (2007). *Evolution et situation actuelle de l'élevage au Cameroun : Rapport de synthèse*. Cameroon	Y	Y	Y	Y	Y	Y	Y	Y	High
37.	Bronsvoort, B. M. C., Anderson J., Corteyn A., Hamblin P., Kitching R. P., Nfon C., & Morgan K. L. (2006). Geographical and age-stratified distributions of foot-and-mouth disease virus-seropositive and probang-positive cattle herds in the Adamawa province of Cameroon. *Veterinary Record* (159), 299-308.	Y	Y	Y	Y	Y	Y	Y	Y	High
38.	Bronsvoort, B. M. C., Radford A. D., Tanya V. N., Nfon C., Kitching R. P., & Morgan K. L. (2004). Molecular Epidemiology of Foot-and-Mouth Disease Viruses in the Adamawa Province of Cameroon. *Journal of Clinical Microbiology, 42*(5), 2186–2196.	Y	Y	U	Y	N	Y	Y	Y	High
39.	Bronsvoort, B. M. C., Tanya V. N., Kitching R. P., Nfon C., Hamman S. M., & Morgan K. L. (2003). Foot and Mouth Disease and Livestock husbandry practices in the Adamawa province of Cameroon. *Tropical Animal Health and Production,* 35, 491-507.	Y	Y	U	Y	N	Y	Y	Y	High
40.	Chahdini, G. A., Mamoudou A., Pagou K. H., Abah S., Woi M., & Mpoame M. (2020). Hepatic and Rumenal Worms Infestations of Cattle in Vina Division (Adamawa – Cameroon). *International Journal of Forest, Animal and Fisheries Research, 4*(1), 14-28; https://dx.doi.org/10.22161/ijfaf.4.1.2	Y	Y	Y	Y	Y	Y	Y	Y	High

41.	Chasar, A., Loiseau, C., Valkiūnas, G., Iezhova, T., Smith, T. B., & Sehgal, R. N. (2009). Prevalence and diversity patterns of avian blood parasites in degraded African rainforest habitats. *Molecular ecology*, *18*(19), 4121–4133. https://doi.org/10.1111/j.1365-294X.2009.04346.x	Y	N	Y	Y	N	Y	U	Y	Medium
42.	Chollet, J.-Y., Jacquiet Ph., Cardinale E., Ndamkou-Ndamkou C., Diop C. , A., T., & Dorchies P., . (2000). Cooperia pectinata and C. punctata, parasites of the abomasum of cattle in northern Cameroon (Central Africa). *Veterinary Parasitology*, 88(1-2), 135–138. https://doi.org/10.1016/s0304-4017(99)00202-2	Y	Y	Y	I	I	Y	Y	Y	High
43.	Dicko, P. S. (2015). *Prévalence et facteurs de risque des parasites gastro-intestinaux des bovins dans le ranch de Ndawara région du Nord-Ouest Cameroun [Unpublished doctoral dissertation].* (Doctor of Veterinary Medicine), Institut Supérieur des Sciences de la Santé, Université des Montagnes, Bangangté, Cameroon.	N	Y	Y	Y	Y	Y	Y	Y	High
44.	Diezoumbe, W. R. (2017). *Prévalence et facteurs de risque des hémoparasites des petits ruminants abattus dans la ville de Ngaoundéré [Unpublished doctoral dissertation].* (Doctor of Veterinary Medicine), School of Veterinary Medicine and Sciences, University of Ngaoundere, Cameroon.	Y	Y	U	Y	Y	Y	Y	Y	High
45.	Djiojo, T. A. B. (2019). *Prévalence et facteurs favorisant des parasites gastro-intestinaux chez le porc dans le département de la Menoua (Cameroun) [Unpublished doctoral dissertation].* (Doctor of Veterinary Medicine), Institut Supérieur des Sciences de la Santé, Université des Montagnes, Bangangté, Cameroon.	Y	Y	U	Y	Y	Y	Y	Y	High
46.	Djonmaila, D. J. (2016). *La co-endémicité des cysticercoses et des cœnuroses ovines, caprines et porcines dans le Département de la Bénoué, Région du Nord-Cameroun [Unpublished doctoral dissertation].* (Doctor of Veterinary Medicine), School of Veterinary Medicine and Sciences, Cameroon.	N	Y	Y	Y	Y	Y	Y	Y	High
47.	Djuidje, K. M. (2019). *Prévalence de la Peste Porcine Africaine et de la Salmonellose dans les élevages à l'Ouest-Cameroun [Unpublished doctoral dissertation].* (Doctor of Veterinary Medicine), Institut Supérieur des Sciences de la Santé, Université des Montagnes, Bangangté, Cameroon.	Y	Y	Y	Y	Y	Y	Y	Y	High
48.	Djuikwo-Teukeng, F. F. D., Simo A. K., Allienne J-F., Rey O., Ngapagna A. N., Tchuem-Tchuente L. A., & Boissier J. (2019). Population genetic structure of Schistosoma bovis in Cameroon. *Parasites & Vectors*, 12(1), 56. https://doi.org/10.1186/s13071-019-3307-0	Y	Y	U	N	Y	Y	Y	Y	High
49.	Dongmo, B. J. (2016). *Séroprévalence et facteurs de risque de la fièvre aphteuse chez les porcs dans la région de l'Extreme-Nord Cameroun [Unpublished doctoral dissertation].* (Doctor of Veterinary Medicine), Schol of Veterinary Medicine and Sciences, University of Ngaoundere, Cameroon.	Y	Y	Y	Y	Y	Y	Y	Y	High
50.	Dongmo, B. J. (2017). *Prevalence des tiques et hemoparasites transmis par les tiques chez les petits ruminants dans le departement du Dja et lobo (region du sud cameroun) [Unpublished doctoral dissertation]*. MSc Dissertation. faculte d'Agronomie et des Sciences Agricoles, University of Dschang. Cameroon.	Y	Y	Y	Y	Y	Y	Y	Y	High

51.	Dongmo, N. R. (2019). *Epidémiologie de la cysticercose porcine dans le département de la Menoua (Ouest-Cameroun) [Unpublished doctoral dissertation].* (Doctor in Veterinary Medicine), School of Veterinary Medicine and Sciences, University of Ngaoundere, Cameroon.	Y	Y	Y	Y	Y	Y	Y	Y	High
52.	Egbe, N. F., Muwonge A., Ndip L., Kelly R. F., Sander M., Tanya V., & Bronsvoort B. M. C. (2017). Molecular epidemiology of Mycobacterium bovis in Cameroon. *Nature: scientific reports,* 7(1), 4652. https://doi.org/10.1038/s41598-017-04230-6	Y	Y	Y	Y	N	Y	N	Y	High
53.	Egbe, N. F., Muwonge A., Ndip L., Kelly R. F., Sander M., Tanya V., & Bronsvoort B. M. C. (2016). Abattoir-based estimates of mycobacterial infections in Cameroon. *Nature scientific reports*, 6, 24320. https://doi.org/10.1038/srep24320	Y	Y	Y	Y	N	Y	N	Y	High
54.	Ekoual, K. J. A. (2018). *Effets anthelminthiques comparés de : Lophira lanceolata (dalhedji) et Vitellaria paradoxa (Kalhedji) à ceux de l'albendazole sur les helminthes gastro-intestinaux des zébus dans la zone soudanà-guinéenne (Adamaoua-Cameroun) [Unpublished doctoral dissertation].* (Doctor of Veterinary Medicine), School of Veterinary Medicine and sciences, University of Ngaoundere, Cameroon.	Y	Y	N	Y	Y	Y	N	Y	High
55.	Esemu, S. N., Ndip R. N., & Ndip L. M. (2018). Detection of Ehrlichia ruminantium infection in cattle in Cameroon. *BMC Research Notes,* 11(1), 388. https://doi.org/10.1186/s13104-018-3479-2	Y	Y	I	U	N	Y	Y	Y	High
56.	Fogue, P. S., Njiokou F., & Simo G. (2017). Genetic structure of Trypanosoma congolense "forest type" circulating in domestic animals and tsetse flies in the South-West region of Cameroon. *Parasite, 24*, 51. https://doi.org/10.1051/parasite/2017052	Y	I	I	Y	Y	Y	I	Y	High
57.	Fotso, M. N. G. (2014). *Etude lésionnelle de la Distomatose bovine à l'abattoir de Yaoundé [Unpublished doctoral dissertation].* (Doctor in Veterinary Medicine) , Institut Supérieur des Sciences de la Santé, Université des Montagnes, Bangangté, Cameroon	Y	Y	Y	Y	Y	Y	Y	Y	High
58.	Francioli, K. K., Bouba, Foko E., Ngatchou A. F., Eyangoh S., & Etoa F-X. (2013). First Insight into the Current Prevalence of Bovine Tuberculosis in Cattle Slaughtered in Cameroon: the Case of Main Abattoirs of Yaoundé and Douala. *British Microbiology Research Journal, 3*(3), 272-279.	Y	Y	U	Y	Y	Y	Y	Y	High
59.	Garin, B., Gouali, M., Wouafo, M., Perchec, A. M., Pham, M. T., Ravaonindrina, N., Urbès, F., Gay, M., Diawara, A., Leclercq, A., Rocourt, J., & Pouillot, R. (2012). Prevalence, quantification and antimicrobial resistance of Campylobacter spp. on chicken neck-skins at points of slaughter in 5 major cities located on 4 continents. *International journal of food microbiology, 157*(1), 102–107. https://doi.org/10.1016/j.ijfoodmicro.2012.04.020	Y	Y	N	Y	Y	Y	U	Y	High
60.	Haman, Z. (2019). *Séroprévalence et facteurs de risque de la Pleuropneumonie Contagieuse Caprine (PPCC) dans la région du Nord-Cameroun [Unpublished doctoral dissertation].* (Doctor of Veterinary Medicine), School of Veterinary Medicine and Sciences, University of Ngaoundere, Cameroon.	Y	Y	Y	Y		Y	Y	Y	High
61.	Handel, I. G., Willoughby, K., Land, F., Koterwas, B., Morgan, K. L., Tanya, V. N., & Bronsvoort, B. M. (2011). Seroepidemiology of bovine viral diarrhoea virus (BVDV) in the Adamawa Region of Cameroon and use of the SPOT test to identify herds with PI calves. *PloS one*, *6*(7), e21620. https://doi.org/10.1371/journal.pone.0021620	N	Y	U	U	N	Y	N	U	Low
62.	Ikoum, D. (2017). *Infestation et prévalence de la trypanosomose bovine dans le ranch de la SODEPA de Ndokayo [Unpublished doctoral dissertation].* (Doctor of Veterinary Medicine), School of Veterinary Medicine and Sciences, Cameroon.	Y	Y	Y	Y	Y	Y	Y	Y	High

63.	Kelly, R. F., Callaby, R., Egbe, N. F., Williams, D., Victor, N. N., Tanya, V. N., Sander, M., Ndip, L., Ngandolo, R., Morgan, K. L., Handel, I. G., Mazeri, S., Muwonge, A., & de C Bronsvoort, B. M. (2018). Association of *Fasciola gigantica* Co-infection With Bovine Tuberculosis Infection and Diagnosis in a Naturally Infected Cattle Population in Africa. *Frontiers in veterinary science, 5*, 214. https://doi.org/10.3389/fvets.2018.00214	Y	Y	Y	Y	Y	Y	N	Y	High
64.	Kelly, R. F., Mazeri S., Hartley C., Hamman S. M., Ngu N. V., Nkongho E. F., D. J. L., & Williams D. J. L. (2019). Assessing the performance of a Fasciola gigantica serum antibody ELISA to estimate prevalence in cattle in Cameroon. *BMC Veterinary Research*, 15(1), 8. https://doi.org/10.1186/s12917-018-1762-z	Y	Y	Y	Y	Y	Y	Y	Y	High
65.	Kenton, L.M., Handel, I. G., Tanya, V. N., Hamman, S. M., Nfon, C., Bergman, I. E., Malirat, V., Sorensen, K. J., & de C Bronsvoort, B. M. (2014). Accuracy of herdsmen reporting versus serologic testing for estimating foot-and-mouth disease prevalence. *Emerging infectious diseases*, 20(12), 2048–2054. https://doi.org/10.3201/eid2012.140931	Y	Y	Y	Y	Y	Y	Y	Y	High
66.	Kochakbe, C. G. (2016). *Dissémination de salmonella Spp de l'abattoir municipal de Maroua.* (Doctor of veterinary Medicine), School of veterinary Medicine and Sciences, University of Ngaoundezre, Cameroon.	Y	Y	N	Y	N	Y	Y	Y	High
67.	Kong, A. T., Munji V. N., Itoe S. M., Hako A. T., & Leinyuy I. (2016). Seroprevalence of Brucella abortus in the Bamenda municipal abattoir of the Western Highlands of Cameroon. *Greener Journal of Agricultural Sciences 6*(8), 245-251. http://doi.org/210.15580/GJAS.12016.15588.082216135.	Y	Y	U	Y	Y	Y	Y	Y	High
68.	Kouam, K. M., Biekop, H. M. F., Katte, B., & Teguia, A. (2019). Risk factors of Salmonella infection in laying hens in Menoua Division, Western region of Cameroon (Central Africa). *Comparative Immunology, Microbiology and Infectious Diseases*, 67, 101370. https://doi.org/10.1016/j.cimid.2019.101370	Y	Y	U	Y	N	Y	Y	Y	High
69.	Kouam, M. K., & Dongmo, J. B. (2018). Factors affecting tick infestations in small ruminants under extensive system in Dja et Lobo in the South Region of Cameroon (Central Africa). *Veterinary Parasitology: Regional Studies and Reports, 14*, 131-136.	Y	Y	Y	Y	Y	Y	Y	Y	High
70.	Kouam, M. K., Meningue, R., & Fon, D. E. (2019). Parasitic causes of organ condemnation in cattle slaughtered in Fako abattoirs, South-West region of Cameroon, and estimate of financial losses. *Journal of helminthology, 93*(3), 367–371. https://doi.org/10.1017/S0022149X18000391	Y	Y	Y	Y	N	Y	Y	Y	High
71.	Kouam, M. K., Ngueguim F. D., & Kantzoura V. (2018). Internal Parasites of Pigs and Worm Control Practices in Bamboutos, Western Highlands of Cameroon. *Journal of Parasitology Research*,10, 8242486. https://doi.org/10.1155/2018/8242486	Y	Y	Y	Y	N	Y	Y	Y	High
72.	Kouam, M. K., Ngueguim F. D., & Kantzoura V. (2018). Internal Parasites of Pigs and Worm Control Practices in Bamboutos, Western Highlands of Cameroon. *Journal of Parasitology Research*, 10. https://doi.org/10.1155/2018/8242486.	Y	Y	Y	Y	Y	Y	Y	Y	High
73.	Kouam, S. A. M. (2015). *Epidémiologie de la gale sarcoptique dans les élevages de porcs du département de la Mifi (Ouest cameroun).* (Doctor of Veterinary Medicine), Institut Supérieur des Sciences de la Santé, Université des Montagnes, Bangangté, Cameroon.	Y	Y	Y	Y	Y	Y	Y	Y	High
74.	Kouengoua, K. A. P. (2014). *Etude des lésions de la tuberculose bovine à l'abattoir de Yaoundé [Unpublished doctoral dissertation].* (Doctor of Veterinary Medicine), Institut Supérieur des Sciences de la Santé, Université des Montagnes, Bangangté, Cameroon.	Y	Y	Y	Y	Y	Y	Y	Y	High

75.	Kouonmo, N. L. (2016). *Séroprévalence et facteurs de risue de la brucellose bovine et humaine dans la ville de Ngaoundéré [Unpublished doctoral dissertation].* (Doctor of Veterinary Medicine), School of Veterinary Medicine and Sciences, University of Ngaoundere, Cameroon.	Y	Y	Y	Y	Y	Y	Y	Y	High
76.	Kuate, W. B. V. (2016). *Prévalence et facteurs de risque par les éleveurs de la fièvre aphteuse chez les bovins dans les départements de la Vina, du Diamare et du Logone-et-Chari [Unpublished doctoral dissertation].* (Doctor of Veterinary Medicine), School of Veterinary Medicine and Sciences, University of Ngaoundere, Cameroon.	Y	Y	N	N	Y	Y	Y	Y	High
77.	Larison, B., Njabo, K. Y., Chasar, A., Fuller, T., Harrigan, R. J., & Smith, T. B. (2014). Spillover of pH1N1 to swine in Cameroon: an investigation of risk factors. *BMC veterinary research, 10*, 55. https://doi.org/10.1186/1746-6148-10-55	Y	Y	U	I	Y	Y	N	Y	High
78.	LeBreton, M., Umlauf S Djoko C. F., Peter Daszak, D. S. B., ¶, Paul Yemgai Kwenkam, & Wolfe, a. N. D. (2006). Rift Valley Fever in Goats, Cameroon. *Emerging Infectious Diseases, 12*(4), 702-703.	Y	Y	U	U	Y	N	Y	N	Medium
79.	Lewong, H. H. (2016). *Prévalences des mammites subcliniques chez les vaches en lactation dans la ville de Ngaoundéré et ses environs [Unpublished doctoral dissertation].* (Doctor of Veterinary Medicine), School of Veterinary Medicine and Sciences, University of Ngaoundere, Cameroon.	Y	Y	Y	Y	Y	Y	Y	Y	High
80.	Loyem, F. E. (2019). *Comparison of serological status and occurrence of reproductive disordes in Brucella positive and negative herds in Vina division, adamawa region (Cameroun) [Unpublished doctoral dissertation].* (Doctor in Veterinary Medicine), School of Veterinary Medicine and Sciences, University of Ngaoundere, Cameroon.	Y	Y	I	Y	Y	Y	I	Y	High
81.	Loyem, F. E. (2019). *Comparison of serological status and occurrence of reproductive disordes in Brucella positive and negative herds in Vina division, adamawa region (Cameroun) [Unpublished doctoral dissertation].* (Doctor in Veterinary Medicine), School of Veterinary Medicine and Sciences, University of Ngaoundere, Cameroon.	Y	Y	U	Y	N	Y	I	Y	High
82.	Ludi, A., Ahmed, Z., Pomeroy, L. W., Pauszek, S. J., Smoliga, G. R., Moritz, M., Dickmu, S., Abdoulkadiri, S., Arzt, J., Garabed, R., & Rodriguez, L. L. (2016). Serotype Diversity of Foot-and-Mouth-Disease Virus in Livestock without History of Vaccination in the Far North Region of Cameroon. *Transboundary and emerging diseases, 63*(1), e27–e38. https://doi.org/10.1111/tbed.12227	Y	Y	Y	Y	N	Y	N	Y	High
83.	Mamoudou A., Salhine R., Sevidzem S. L., Achukwi M. D., & Garabed R. (2018). Efficacy of Albendazole on Gastro-Intestinal Strongyles of Catle in Ngaoundere (Adamawa-Cameroon). *Integrative Journal of Veterinary Biosciences, 2*(2), 1-6.	Y	Y	U	U	Y	Y	I	Y	High
84.	Mamoudou, A., Ebene N. J., Suh P. F., & Mfopit M. Y. (2015). Prevalence and impact of bovine trypanosomiasis in Mayo Rey division, a Soudano-Sahelian zone of Cameroon. *Journal of Parasitology and Vector Biology 7*(5), 80-88.	Y	Y	U	Y	Y	Y	Y	Y	High
85.	Mamoudou, A., Khan P. V., & Lendzele S. S. (2015). Current prevalence of cattle trypanosomiasis and of its vector in Alme, the infested zone of Adamawa plateau Cameroon, two decades after the tsetse eradication campaign. *International Journal of Biological and Chemical Sciences, 9*(3), 1588-1598.	Y	Y	U	Y	Y	Y	Y	Y	High
86.	Mamoudou, A., Mbakou L. M., Ngu Ngwa V., Sevidzem S. L., Zoli A. P., & D., A. M. (2016). Preliminary assesment of bovine trypanosomiasis and its vectors in Santa, Bali and Bafut Sub-divisions of the North West Region, Cameroon. *International Journal of Biological and Chemical Sciences, 10*(1), 1-12.	Y	Y	Y	Y	Y	Y	Y	Y	High

87.	Mamoudou, A., Nguetoum N. C., Zoli P. A., & Sevidzem S. L. (2016). Identification and Infestation of Ticks on Cattle in the Peri-Urban zone of Ngaoundere. *Journal of Veterinary Science & Medical Diagnosis* , *5*(4), doi: 10.4172/2325-9590.1000207.	Y	Y	Y	Y	N	Y	N	Y	High
88.	Mamoudou, A., Nguetoum, N. C., Sevidzem, S. L., Manchang, T. K., Ebene, N. J., & Zoli, P. A. (2017). Bovine babesiosis and Anaplasmosis in Some Cattle Farms in the Vina Division, Adamaoua Plateau. *International Journal of Livestock Research, 6*, 69-80. Doi: 10.5455/ijlr.20170423025510.	Y	Y	Y	Y	Y	Y	Y	Y	High
89.	Mamoudou, A., Njanloga, A., Hayatou, A., Suh, P. F., & Achukwi, M. D. (2016). Animal trypanosomosis in clinically healthy cattle of north Cameroon: epidemiological implications. *Parasites & vectors*, *9*, 206. https://doi.org/10.1186/s13071-016-1498-1	Y	Y	U	Y	Y	Y	Y	Y	High
90.	Mamoudou, A., Sakativa D., Ebene N. J., & Z.oli P. A., (2016). Effet de l'albendazole sur les helminthes gastro-intestinaux et la productivité des jeunes bovins de l'Adamaoua Cameroun. *Tropicultura*, *34*(2), 140-149.	Y	N	U	N	Y	Y	Y	Y	High
91.	Mamoudou, A., Suh P. F., & Ebene N. J. (2015). Trypanosomes and helminths infections in Mayo Rey Division of Cameroon and impact of concurrent infections on cattle. *Journal of Veterinary Medicine and Animal Health*, *7*(6), 215-220.	Y	Y	U	Y	Y	Y	Y	Y	High
92.	Mamoudou, A., Zoli A., Mbahin N., Tanenbe C., Bourdanne A., Clausen P. H., & Geerts S. (2006). Prevalence and incidence of bovine trypanosomosis on the Adamaoua plateau in Cameroon 10 years after the tsetse eradication campaign. *Veterinary Parasitology, 142*, 16-22.	Y	Y	U	Y	N	Y	Y	Y	High
93.	Mamoudou, A., Zoli P. A., & Tchoua P. (2009). Parasitological prevalence of bovine trypanosomosis in the Faro and Deo division valley of the Adamaoua plateau, Cameroon. *International Journal of Biological and Chemical Sciences*, *3*(5), 1192-1197.	Y	Y	Y	Y	Y	Y	Y	Y	High
94.	Mamoudou, A., Zoli P. A., Tanenbe C., Andrikaye J. P., Bourdanne, Marcotty T., Delespaux V., Clausen P.-H., &Geerts S. (2006). Evaluation sur le terrain et sur souris de la résistance des trypanosomes des bovins du plateau de l'Adamaoua au Cameroun à l'acéturate de diminazène et au chlorure d'isométamidium. *Revue d'élevage et de médecine vétérinaire des pays tropicaux.*, *59*(1-4), 11-16. https://doi.org/10.19182/remvt.9948	Y	Y	U	Y	Y	Y	Y	Y	High
95.	Manchang, T. K., Mfopit M. Y., Awah-Ndukum J., Nguini A. J., Soh B. G., & Bayemi P.H. (2016). Seasonal Evaluation of Some Disease Conditions of Cattle Slaughtered In Ngaoundere-Municipal Abattoir, Cameroon. *International Journal of Livestock Research*, *6*(11), 46-53. doi: 10.5455/ijlr.20160919115434.	Y	Y	Y	Y	N	Y	N	Y	High
96.	Manglang, T. (2019). *Séroprévalence et facteurs de risque de la Peste des Petits Ruminants chez les ovins des régions de l'adamaoua et du Nord Cameroun. [Unpublished doctoral dissertation]* (Doctor in Veterinary Medicine), School of Veterinary Medicine and Sciences, University of Ngaoundere, Cameroon.	Y	Y	Y	Y	Y	Y	Y	Y	High
97.	Mbiakop, N. J. (2018). *Prévalence et intensité des parasites gastro-intestinaux des petits ruminants dans le département du Ndé [Unpublished doctoral dissertation].* (Doctor in Veterinary Medicine), Institut Supérieur des Sciences de la Santé, Université des Montagnes, Bangangté, Cameroon.	Y	Y	Y	Y	Y	Y	Y	Y	High
98.	Mbitkebeyo, P. C. (2018). *Prévalence et facteurs de risque associés à la coccidiose dans les élevages de poulets de chair dans le département du Ndé [Unpublished doctoral dissertation].* (Doctor in Veterinary Medicine), Institut Supérieur des Sciences de la Santé, Université des Montagnes, Bangangté, Cameroon.	Y	Y	Y	Y	Y	Y	Y	Y	High

99.	Mbuh J. V., Ndamukong K. J. N., Ntonifor N., & Nforlem G. F. (2008). Parasites of sheep and goats and their prevalence in Bokova, a rural area of Buea Sub Division, Cameroon. *Veterinary Parasitology 156*, 350–352.	N	Y	Y	Y	N	Y	I	Y	High
100.	Metou, O. M. N. (2016). *Influence de certains paramètres sur les prévalences des parasitoses gastro-intestinales des porcs dans les élevages traditionnels de trois départements de la région de l'Extreme-Nord Cameroun [Unpublished doctoral dissertation].* (Doctor of Veterinary Medicine), School of Veterinary Medicine and Sciences, University of Ngaoundere, Cameroon.	Y	Y	Y	Y	Y	Y	Y	Y	High
101.	MINEPIA, FAO, & OMC. (2016). *Plan stratégique de prévention et de lutte contre la peste des Petits Ruminants (PPR) au Cameroun*. Retrieved from Cameroon:	Y	Y	Y	Y	N	Y	N	Y	High
102.	Modiyinji, A. F., Atsama M. A., Monamele G. C., Nola M., & Njouom R. (2018). High seroprevalence of hepatitis E among pigs suggests an animal reservoir in Cameroon. *The Journal of Infection in Developping Countries*, *12*(8), 676-679.	Y	Y	U	Y	Y	Y	I	Y	High
103.	Modjo, K. E. L. (2014). *Séroprévalence de la fièvre aphteuse chez les Bovins dans la région du Nord Cameroun [Unpublished doctoral dissertation].* (Doctor of Veterinary Medicine), Institut Supérieur des Sciences de la Santé, Université des Montagnes, Bangangté, Cameroon.	Y	Y	Y	Y	Y	Y	Y	Y	High
104.	Mohamadou, B. (2017). *Etude de la prévalence de la trypanosomose chez les bovins abattus à l'abattoir municipal de Ngaoundéré (Cameroun) [Unpublished doctoral dissertation].* (Doctor of Veterinary Medicine), School of Veterinary Medicine and Sciences, University of Ngaoundere, Cameroon.	Y	Y	Y	Y	Y	Y	Y	Y	High
105.	Mohamadou, B. (2017). *Etude de la prévalence de la trypanosomose chez les bovins abattus à l'abattoir municipal de Ngaoundéré (Cameroun) [Unpublished doctoral dissertation].* (Doctor of Veterinary Medicine), School of Veterinary Medicine and Sciences, University of Ngaoundere, Cameroon.	Y	Y	Y	Y	Y	Y	Y	Y	High
106.	Moiffo, K. A. M. (2016). *Dépistage des anticorps anti-brucella chez les porcs dans certaines régions du Cameroun. [Unpublished doctoral dissertation].* (Doctor of Veterinary Medicine), School of Veterinary Medicine and Sciences, University of Ngaoundere, Cameroon.	Y	N	Y	Y	Y	Y	N	Y	High
107.	Mpouam, S. E., Achukwi M. D., Feussom K. J. M., Bengaly Z., & Ouedraogo G. A. (2011). Serological and parasitological prevalence of bovine trypanosomosis in small holder farms of the Vina division, Adamawa region of Cameroon. *Journal of Parasitology and Vector Biology, 3*(4), 44-51.	Y	N	U	Y	Y	Y	Y	Y	High
108.	Musallam, I., Ndour, A. P., Yempabou, D., Ngong, C. C., Dzousse, M. F., Mouiche-Mouliom, M. M., Feussom, J., Ntirandekura, J. B., Ntakirutimana, D., Fane, A., Dembele, E., Doumbia, A., Ayih-Akakpo, A., Pato, P., Pali, M., Tapsoba, A., Compaore, G. M., Gagara, H., Garba, A. I., Chengat Prakashbabu, B., … Guitian, J. (2019). Brucellosis in dairy herds: A public health concern in the milk supply chains of West and Central Africa. *Acta tropica*, *197*, 105042. https://doi.org/10.1016/j.actatropica.2019.105042	Y	Y	U	I	Y	Y	U	Y	High
109.	Ndze, V. N., Cadar, D., Cságola, A., Kisfali, P., Kovács, E., Farkas, S., Ngu, A. F., Esona, M. D., Dán, Á., Tuboly, T., & Bányai, K. (2013). Detection of novel porcine bocaviruses in fecal samples of asymptomatic pigs in Cameroon. *Infection, genetics and evolution: journal of molecular epidemiology and evolutionary genetics in infectious diseases*, *17*, 277–282. https://doi.org/10.1016/j.meegid.2013.03.006	Y	I	I	I	N	Y	I	Y	High

110.	Ngadjie, T. M. (2019). *Prévalence et facteurs favorisants associés à la coccidiose dans les élevages de poulets de chair du Koung-khi [Unpublished doctoral dissertation]*. (Doctor of Veterinary Medicine), Institut Supérieur des Sciences de la Santé, Université des Montagnes, Bangangté, Cameroon.	Y	Y	U	U	Y	Y	Y	Y	High
111.	Nghonjuyi, N. W., Kimbi H. K., & Tiambo C. K. (2014). Study of Gastro–Intestinal Parasites of Scavenging Chickens in Fako Division, Southwest Cameroon. *The Journal of Advances in Parasitology 1*(2), 30-34.	Y	Y	Y	U	N	Y	Y	Y	High
112.	Ngole, I. U., Ndamukong, K. J., & Mbuh, J. V. (2003). Internal parasites and haematological values in cattle slaughtered in Buea subdivision of Cameroon. *Tropical animal health and production*, *35*(5), 409–413. https://doi.org/10.1023/a:1025811428008	Y	N	U	U	U	U	U	U	Low
113.	Ngomtcho, S., Weber, J. S., Ngo Bum, E., Gbem, T. T., Kelm, S., & Achukwi, M. D. (2017). Molecular screening of tsetse flies and cattle reveal different Trypanosoma species including T. grayi and T. theileri in northern Cameroon. *Parasites & vectors*, *10*(1), 631. https://doi.org/10.1186/s13071-017-2540-7	Y	Y	Y	U	N	Y	N	Y	High
114.	Ngoudjou, M. S. V. (2018). *Prévalence des lésions tuberculeuses viscérale à l'abattoir de Douala [Unpublished doctoral dissertation]*. (Doctor in Veterinary Medicine), Institut Supérieur des Sciences de la Santé, Université des Montagnes, Bangangté, Cameroon.	Y	Y	Y	Y	Y	Y	Y	Y	High
115.	Ngounou, D. Y. D. (2015). *Séroprévalence et facteurs de risque de la toxoplasmose chez les ovins dans la localité de Ndawara (Nord-Ouest, Cameroun) [Unpublished doctoral dissertation]*. (Doctor of Veterinary Medicine), Institut Supérieur des Sciences de la Santé, Université des Montagnes, Bangangté, Cameroon.	Y	Y	Y	Y	Y	Y	Y	Y	High
116.	Nguena, G. N. R. (2017). *Epidémiolgoie de la peste porcine africaine dans les régions septentrionales du Cameroun [Unpublished doctoral dissertation]*. (Doctor of veterinary Medicine), School of Veterinary Medicine and Sciences, University of Ngaoundere, Cameroon.	Y	Y	Y	Y	Y	Y	Y	Y	High
117.	Nguetoum, N. C. (2016). *Inventaire des espèces de tiques et Prévalence des Hémoparasites transmis par ces dernières en zone périurbaine de Ngaoundéré [Unpublished doctoral dissertation]*. (Doctor in Veterinary Medicine), School of Veterinary Medcine and Sciences, University of Ngaoundere, Cameroon.	Y	Y	Y	U	N	Y	Y	Y	High
118.	Ngu-Ngwa, V., Bessong-Takang N. A., & Awah-Ndukum J. (2020). Seroprevalence and Risk Factors of Leptospirosis among Slaughtered Cattle and Abattoir Workers in Ngaoundéré, Cameroon. *Asian Journal of Research in Animal and Veterinary Sciences*, *5*(1), 8-19.	Y	Y	Y	Y	Y	Y	Y	Y	High
119.	Ngwing, N. A., Poné, J. W., Mbida, M., Pagnah, A. Z., Njakoi, H., & Bilong, C. F. (2012). A preliminary analysis of some epidemiological factors involved in porcine cysticercosis in Bafut and Santa subdivisions, North West Region of Cameroon. *Asian Pacific journal of tropical medicine*, *5*(10), 814–817. https://doi.org/10.1016/S1995-7645(12)60149-7	Y	Y	Y	U	N	Y	Y	Y	High
120.	Nimpaye, H., Njiokou F., Njine T., Njitchouang G. R., Cuny G., Herder S., Simo G. (2011). Trypanosoma Vivax, T. Congolense "Forest type" and T. Simaie : Prevalence in domestic animals of sleeping sickness in Cameroon. *Parasite: Journal de la Société Française de Parasitologie*, *18*, 171-179.	Y	Y	U	Y	Y	Y	N	Y	High
121.	Njabo, K. Y., Fuller, T. L., Chasar, A., Pollinger, J. P., Cattoli, G., Terregino, C., Monne, I., Reynes, J. M., Njouom, R., & Smith, T. B. (2012). Pandemic A/H1N1/2009 influenza virus in swine, Cameroon, 2010. *Veterinary microbiology*, *156*(1-2), 189–192. https://doi.org/10.1016/j.vetmic.2011.09.003	Y	Y	U	Y	Y	Y	N	Y	High

122.	Njanja, D. G. W. (2018). *Prévalence, étiologie et facteurs de risque de l'endométrite chez les zébus (Bos indicus) á l'abattoir municipal de Ngaoundéré, région de l'Adamaoua Cameroun [Unpublished doctoral dissertation].* (Doctor of Veterinary Medicine), School of Veterinary Medicine and Sciences, University of Ngaoundere, Cameroon.	Y	Y	Y	Y	Y	Y	Y	Y	High
123.	Njongmeta, N. L., Tanya V. N., & Keng A. (2001). Enquête sur les sérotypes de Pasteurella multocida dans les zones de savane du Cameroun. *Revue d'élevage et de médecine vétérinaire des pays tropicaux*, *54*(3-4), 187-189.	N	Y	U	Y	N	Y	I	Y	High
124.	Nkana, K. J. G. (2014). *Epidémiologie de la PPA dans les élevages porcins de la ville de Bafoussam (Ouest du Cameroun) [Unpublished doctoral dissertation].* (Doctor in Veterinary Medicine), Institut Supérieur des Sciences de la Santé, Université des Montagnes, Bangangté, Cameroon.	Y	Y	Y	Y	Y	Y	Y	Y	High
125.	Nkinin, S. W., Njiokou F., Penchenier L., Grebaut P., Simo G., & Herder S. (2002). Characterization of Trypanosoma brucei s.l. subspecies by isoenzymes in domestic pigs from the Fontem sleeping sickness focus of Cameroon. *Acta Tropica*, *81*, 225–232.	N	Y	U	U	N	Y	Y	Y	High
126.	Nsangou, N. S. (2017). *Séroprévalence et facteurs de risque de la fièvre aphteuse chez les petits ruminants domestiques dans la région du Nord Cameroun [Unpublished doctoral dissertation].* (Doctor of Veterinary Medicine), School of Veterinary Medicine and Sciences, University of Ngaoundere, Cameroon.	Y	Y	Y	Y	Y	Y	Y	Y	High
127.	Nsun, A. A. (2019). *Prevalence and perception of the risk of trichinellosis in pugs destined for human consumption in Douala, Cameroon [Unpublished doctoral dissertation].* (Doctor of Veterinary Medicine), School of Veterinary Medicine and Sciences, University of Ngaoundere, Cameroon.	Y	Y	Y	Y	Y	Y	Y	Y	High
128.	Ntonifor, H. N., Shei S. J., Ndaleh N. W., & Mbunkur G. N. (2013). Epidemiological studies of gastrointestinal parasitic infections in ruminants in Jakiri, Bui Division, North West Region of Cameroon. *Journal of Veterinary Medicine and Animal Health*, *5*(12), 344-352.	N	Y	U	U	N	Y	N	Y	High
129.	Nzale, N.C.E. (2014). *Contribution à l'étude de quelques parasitoses bovines à l'abattoir de Bangangté [Unpublished doctoral dissertation].* (Doctor in Veterinary Medicine), Institut Supérieur des Sciences de la Santé, Université des Montagnes, Bangangté, Cameroon.	Y	Y	Y	Y	Y	Y	Y	Y	High
130.	Nzouankeu, A., Ngandjio A., Ejenguele G., Njine T., & Wouafo M. N. (2010). Multiple contaminations of chickens with Campylobacter, Escherichia coli and Salmonella in Yaounde (Cameroon). *Journal of Infection in Developing Countries*, *4*(9), 583-686.	Y	Y	Y	Y	N	Y	N	Y	High
131.	Ojong, B. W. (2011). *Situation of brucellosis in beef-type cattle raised under different husbandry systems in Cameroon*. Retrieved from Cameroon:	N	Y	Y	Y	Y	Y	N	Y	High
132.	Ojong, B. W., Saccà E., Bessong P., & Piasentier E. (2016). Prevalence of bovine dermatophilosis and disease-associated alleles in zebu Goudali cattle and their Italian Simmental crosses ranching in the western highland plateau savannah of Cameroon. *Tropical Animal Health and Production*, *48*, 1329–1335.	N	Y	Y	Y	Y	Y	N	Y	High
133.	Ojong, B., Macleod E., Ndip M. L., Zuliani A., & Piasentier E. (2016). Brucellosis in Cameroon: Seroprevalence and risk factors in beef-type cattle. *International Journal of Infectious Diseases*, *53S*, http://dx.doi.org/10.1016/j.ijid.2016.1011.1161.	Y	Y	Y	Y	Y	Y	Y	Y	High
134.	Pagou, K. H. (2019). *Prévalence et facteurs de risque des distomatoses hépatobiliaires bovines dans la zone périurbaine de Ngaoundéré [Unpublished doctoral dissertation].* (Doctor in Veterinary), School of veterinary medicine and Sciences, Cameroon.	Y	Y	Y	Y	Y	Y	Y	Y	High

135.	Paguem, A., Abanda, B., Ndjonka, D., Weber, J. S., Ngomtcho, S., Manchang, K. T., Adoulmoumini, M., Eisenbarth, A., Renz, A., Kelm, S., & Achukwi, M. D. (2019). Widespread co-endemicity of Trypanosoma species infecting cattle in the Sudano-Sahelian and Guinea Savannah zones of Cameroon. *BMC veterinary research*, *15*(1), 344. https://doi.org/10.1186/s12917-019-2111-6	Y	Y	N	Y	Y	Y	Y	Y	High
136.	Pouedet, M. S., Zoli, A. P., Nguekam, Vondou, L., Assana, E., Speybroeck, N., Berkvens, D., Dorny, P., Brandt, J., & Geerts, S. (2002). Epidemiological survey of swine cysticercosis in two rural communities of West-Cameroon. *Veterinary parasitology*, *106*(1), 45–54. https://doi.org/10.1016/s0304-4017(02)00035-3	Y	Y	Y	Y	Y	Y	Y	Y	High
137.	Poueme, R., Stoek, F., Nloga, N., Awah-Ndukum, J., Rissmann, M., Schulz, A., Wade, A., Kouamo, J., Moctar, M., Eisenbarth, A., God-Yang, L., Dickmu, S., Eiden, M., & Groschup, M. H. (2019). Seroprevalence and Associated Risk Factors of Rift Valley Fever in Domestic Small Ruminants in the North Region of Cameroon. *Veterinary medicine international*, *2019*, 8149897. https://doi.org/10.1155/2019/8149897	Y	Y	Y	Y	Y	Y	Y	Y	High
138.	Praet, N., Speybroeck, N., Manzanedo, R., Berkvens, D., Nsame Nforninwe, D., Zoli, A., Quet, F., Preux, P. M., Carabin, H., & Geerts, S. (2009). The disease burden of Taenia solium cysticercosis in Cameroon. *PLoS neglected tropical diseases*, *3*(3), e406. https://doi.org/10.1371/journal.pntd.0000406	N	U	I	I	I	Y	I	I	High
139.	Rissmann, M., Eiden, M., Wade, A., Poueme, R., Abdoulkadiri, S., Unger, H., Ziegler, U., Homeier, T., & Groschup, M. H. (2017). Evidence for enzootic circulation of Rift Valley fever virus among livestock in Cameroon. *Acta tropica*, *172*, 7–13. https://doi.org/10.1016/j.actatropica.2017.04.001	Y	Y	Y	Y	Y	Y	Y	Y	High
140.	S de Paula, V., Wiele, M., Mbunkah, A. H., Daniel, A. M., Kingsley, M. T., & Schmidt-Chanasit, J. (2013). Hepatitis E virus genotype 3 strains in domestic pigs, Cameroon. *Emerging infectious diseases*, *19*(4), 666–668. https://doi.org/10.3201/eid1904.121634	Y	I	I	I	N	Y	N	Y	High
141.	Sakativa, D. (2014). *Evaluer l'Impact de la Vermifugation à l'Albendazole 10% sur le niveau d'excrétion des œufs des helminthes gastro-intestinaux et sur le poids des jeunes bovins des élevages semi-intensifs du Département de la Vina-Région de l'Adamaoua au Cameroun pendant la saison sèche [Unpublished doctoral dissertation]*. (Doctor of Veterinary Medicine), School of Veterinary Medicine and Sciences, University of Ngaoundere, Cameroon.	N	Y	Y	Y	Y	Y	Y	Y	High
142.	Salhine, R. (2016). *Prévalence des helminthes gastro-intestinaux des bovins et effet de l'albendazole sur leur population dans la Vina [Unpublished doctoral dissertation]*. (Doctor in Veterinary Medicine), School of Veterinary Medicine and Sciences, University of Ngaoundere, Cameroon.	Y	Y	U	U	Y	Y	Y	Y	High
143.	Saya, M. (2017). *Séroprévalence et facteurs de risque de la brucellose chez les petits ruminants dans les régions de l'Adamaoua et du Nord-Cameroun [Unpublished doctoral dissertation]*. (Doctor in Veterinary Medicine), School of Veterinary Medicine and Sciences, University of Ngaoundere, Cameroon.	Y	Y	Y	Y	Y	Y	Y	Y	High
144.	Scolamacchia, F., Handel, I. G., Fèvre, E. M., Morgan, K. L., Tanya, V. N., & Bronsvoort, B. M. (2010). Serological patterns of brucellosis, leptospirosis and Q fever in Bos indicus cattle in Cameroon. *PloS one*, *5*(1), e8623. https://doi.org/10.1371/journal.pone.0008623	Y	Y	Y	Y	Y	Y	N	Y	High

145.	Sehgal, R. N. M., Valkiūnas G., Lezhova T. A., & Smith T. B. (2006). Blood parasites of chickens in Uganda and Cameroon with molecular descriptions of Leucocytozoon schoutedeni and Trypanosoma gallinarum. *Journal of Parasitology*, *92*(6), 1336-1343.	Y	Y	U	U	Y	Y	N	Y	High
146.	Sevidzem, S. L., Mamoudou A., Mavoungou J. F., Ikoum D., Lebale M. B. O., Hiol V. D., . . . R., G. (2019). Serological Epidemiology of Foot-and-mouth Disease among Sedentary Mixed-species Herds in Adamawa Region, Cameroon. *Journal of Advances in Microbiology*, *17*(2), 1-14	Y	Y	U	N	Y	Y	Y	Y	High
147.	Shey-Njila, O., Daouda, Nya E., Zoli P. A., Walravens K., Godfroid J., & Geerts S. (2005). Serological Survey of Bovine Brucellosis in Cameroon. *Revue d'élevage et de médecine vétérinaire des pays tropicaux*, *58*(3), 139-143.	Y	Y	Y	Y	Y	Y	N	Y	High
148.	Signie, N. C. (2019). *Prévalence et facteurs de risque des mammites subcliniques dans les élevages de vaches laitière du département du Noun [Unpublished doctoral dissertation].* (Doctor of Veterinary Medicine), Institut Supérieur des Sciences de la Santé, Université des Montagnes, Bangangté, Cameroon.	Y	N	U	Y	Y	Y	N	Y	Medium
149.	Silatsa, B. A., Simo G., Githaka N., Mwaura S., Kamga R. M., Oumarou F., Keambou C., R. P., Djikeng A., Kuiate J-R., Njiokou F., & Pelle R. (2019). A comprehensive survey of the prevalence and spatial distribution of ticks infesting cattle in diferent agro-ecological zones of Cameroon. Parasites& Vectors, 12, 489 . https://doi.org/10.1186/s13071-019-3738-7	Y	I	I	Y	Y	Y	I	Y	High
150.	Silatsa, B. A., Simo, G., Githaka, N., Mwaura, S., Kamga, R. M., Oumarou, F., Keambou, C., Bishop, R. P., Djikeng, A., Kuiate, J. R., Njiokou, F., & Pelle, R. (2019). A comprehensive survey of the prevalence and spatial distribution of ticks infesting cattle in different agro-ecological zones of Cameroon. *Parasites & vectors*, *12*(1), 489. https://doi.org/10.1186/s13071-019-3738-7	Y	Y	Y	Y	Y	Y	Y	Y	High
151.	Simeni, K. F. (2014). *Epidémiologie des parasites gastro-intestinaux des caprins dans le département du Moungo (Littoral Cameroun) [Unpublished doctoral dissertation].* (Doctor in Veterinary Medicine), Institut Supérieur des Sciences de la Santé, Université des Montagnes, Bangangté, Cameroon.	Y	Y	Y	Y	Y	Y	Y	Y	High
152.	Simo, G., Asonganyi T., Nkinin S. W., Njiokou F., & Herder S. (2006). High prevalence of Trypanosoma brucei gambiense group 1 in pigs from the Fontem sleeping sickness focus in Cameroon. *Veterinary parasitology*, *139*, 57-66.	Y	Y	Y	Y	Y	Y	N	Y	High
153.	Simo, G., Fongho P., Farikou O., Ndjeuto-Tchouli N. P. I., Tchouomene-Labou J., Njiokou F., & Asonganyi T. (2015). Trypanosome infection rates in tsetse flies in the “silent” sleeping sickness focus of Bafia in the Centre Region in Cameroon. *Parasites & Vectors*, 8, 528. https://doi.org/10.1186/s13071-015-1156-z	Y	I	I	Y	Y	Y	I	Y	High
154.	Simo, G., Njitchouang G. R., Njiokou F., Cuny G., & Tazoacha A. (2012). Genetic characterization of Trypanosoma brucei circulating in domestic animals of the Fontem sleeping sickness of Cameroon. *Microbes and infection*, *14*, 651-658.	Y	Y	Y	Y	Y	Y	N	Y	High
155.	Simo, G., Sobgwi P. F., Njitchouang G. R., Njiokou F., Kuiate J. R., Cuny G., & Asonganyi T. (2013). Identification and genetic characterization of Trypanosoma congolense in domestic animals in Fontem in the South-West region of Cameroon. *Infection, Genetics and Evolution*, *18*, 66-73.	Y	Y	Y	Y	Y	Y	N	Y	High
156.	Snoeck, C. J., Komoyo, G. F., Mbee, B. P., Nakouné, E., Le Faou, A., Okwen, M. P., & Muller, C. P. (2012). Epidemiology of chicken anemia virus in Central African Republic and Cameroon. *Virology journal*, *9*, 189. https://doi.org/10.1186/1743-422X-9-189	N	Y	I	I	N	Y	Y	Y	High

157.	Snoeck, C. J., Owoade, A. A., Couacy-Hymann, E., Alkali, B. R., Okwen, M. P., Adeyanju, A. T., Komoyo, G. F., Nakouné, E., Le Faou, A., & Muller, C. P. (2013). High genetic diversity of Newcastle disease virus in poultry in West and Central Africa: cocirculation of genotype XIV and newly defined genotypes XVII and XVIII. *Journal of clinical microbiology*, *51*(7), 2250–2260. https://doi.org/10.1128/JCM.00684-13	Y	Y	Y	Y	Y	Y	N	Y	High
158.	Soussai, F. O. (2017). *Prévalences et facteurs de risques des hémoparasites des bovins abattus à l'abattoir municipal de Ngaoundéré [Unpublished doctoral dissertation].* (Doctor of Veterinary Medicine), School of Veterinary Medicine and Sciences, University of Ngaoundere, Cameroon.	Y	Y	Y	Y	Y	Y	Y	Y	High
159.	Suh, P. F., Njiokou F., Mamoudou A., Ahmadou T. M., Mouhaman A., & Garabed R. (2017). Bovine trypanosomiasis in tsetse-free pastoral zone of the Far-North region, Cameroon. *Journal of Vector Borne Diseases*, (54), 263–269.	Y	N	Y	N	Y	Y	N	Y	Medium
160.	Takang, E. E., LeBreton, M., Ayuk, C. E., & MacLeod, E. T. (2019). A socio-economic study of *Fasciola* infections in cattle and sheep at the Etoudi slaughterhouse, Yaoundé, Cameroon. *Journal of helminthology*, *94*, e92. https://doi.org/10.1017/S0022149X19000890	Y	Y	Y	Y	Y	Y	Y	Y	High
161.	Talba, Y. D. (2014). *Etude Epidémiologique de la PPCB dans le Nord Cameroun : Département de la Benoué [Unpublished doctoral dissertation].* (Doctor in Veterinary Medicine), Institut Supérieur des Sciences de la Santé, Université des Montagnes, Bangangté, Cameroon.	Y	Y	Y	Y	Y	Y	Y	Y	High
162.	Tanenbe, C., Gambo H., Musongong A. G., Boris O., & Achukwi M. D. (2010). Prévalence de la trypanosomose bovine dans les départements du Faro et Déo, et de la Vina au Cameroun : bilan de vingt années de lutte contre les glossines. *Revue d'élevage et de médecine vétérinaire des pays tropicaux.*, *63*(3-4), 63-69.	Y	Y	Y	Y	Y	Y	Y	Y	High
163.	Tchedele, A. O. (2017). *Tuberculose bovine chez la chèvre dans la ville de Garoua: performances diagnostiques de l'intradermo-tuberculination, prévalence et facteurs de risque zoonotique [Unpublished doctoral dissertation].* (Doctor of Veterinary Medicine), School of Veterinary Medicine and sciences, University of Ngaoundere, Cameroon.	Y	Y	Y	Y	Y	Y	Y	Y	High
164.	Tchouagoue, E. R. (2014). *Epidémiologie et moyen de lutte contre les parasites gastro- intestinaux chez les caprins dans le Département du Ndé [Unpublished doctoral dissertation].* (Doctor of Veterinary Medicine), Institut Supérieur des Sciences de la Santé, Université des Montagnes, Bangangté, Cameroon.	Y	Y	Y	Y	Y	Y	Y	Y	High
165.	Tchouankui, N. H. (2014). *Epidémiologie des Parasites gastro-intestinaux chez les caprins dans le Département de la Mefou-et-Akono.* (Doctor of Veterinary Medicine), Institut Supérieur des Sciences de la Santé, Université des Montagnes, Bangangté, Cameroon.	Y	Y	Y	Y	Y	Y	Y	Y	High
166.	Tchouankui, N. H. (2014). *Epidémiologie des Parasites gastro-intestinaux chez les caprins dans le Département de la Mefou-et-Akono [Unpublished doctoral dissertation].* (Doctor of Veterinary Medicine), Institut Supérieur des Sciences de la Santé, Université des Montagnes, Bangangté, Cameroon.	Y	Y	Y	Y	Y	Y	Y	Y	High
167.	Tchoumbou, M. A., Mayi, M., Malange, E., Foncha, F. D., Kowo, C., Fru-Cho, J., Tchuinkam, T., Awah-Ndukum, J., Dorazio, R., Nota Anong, D., Cornel, A. J., & Sehgal, R. (2020). Effect of deforestation on prevalence of avian haemosporidian parasites and mosquito abundance in a tropical rainforest of Cameroon. *International journal for parasitology*, *50*(1), 63–73. https://doi.org/10.1016/j.ijpara.2019.10.006	N	Y	U	U	N	Y	U	Y	Low

168.	Tchounkeu, H. C. R. (2017). *Influence du type d'élevage sur la prévalence et l'intensité d'infestation des helminthes gastro-intestinaux et hémoparasites des bovins dans le département de la Vina, zone soudano-guinéenne, Adamaoua-Cameroun [Unpublished doctoral dissertation].* (Doctor of Veterinary Medicine), School of Veterinary Medicine and Sciences, University of Ngaoundere, Cameroon.	Y	Y	Y	Y	Y	Y	N	Y	High
169.	Vondou, L., Zoli, A. P., Nguekam, Pouedet, S., Assana, E., Kamga Tokam, A. C., Dorny, P., Brandt, J., & Geerts, S. (2002). La taeniose/cysticercose à Taenia solium dans la Menoua (Ouest-Cameroun) [Taenia solium taeniasis/cysticercosis in the Menoua division (West Cameroon)]. *Parasite* , *9*(3), 271–274. https://doi.org/10.1051/parasite/2002093271	Y	Y	N	N	Y	Y	N	Y	Medium
170.	Wade, A., Achenbach, J. E., Gallardo, C., Settypalli, T., Souley, A., Djonwe, G., Loitsch, A., Dauphin, G., Ngang, J., Boyomo, O., Cattoli, G., Diallo, A., & Lamien, C. E. (2019). Genetic characterization of African swine fever virus in Cameroon, 2010-2018. Journal of microbiology, 57(4), 316–324. https://doi.org/10.1007/s12275-019-8457-4	N	I	U	U	N	Y	I	Y	High
171.	Wade, A., Jumbo, S. D., Zecchin, B., Fusaro, A., Taiga, T., Bianco, A., Rodrigue, P. N., Salomoni, A., Kameni, J., Zamperin, G., Nenkam, R., Foupouapouognigni, Y., Abdoulkadiri, S., Aboubakar, Y., Wiersma, L., Cattoli, G., & Monne, I. (2018). Highly Pathogenic Avian Influenza A(H5N8) Virus, Cameroon, 2017. *Emerging infectious diseases*, *24*(7), 1367–1370. https://doi.org/10.3201/eid2407.172120	Y	I	I	I	N	Y	I	Y	High
172.	Wade, A., Taiga, Fouda M. A., MaiMoussa A., Feussom K. J. M., Njouom R., . . . G., C. (2018). Highly pathogenic avian influenza A/H5N1 Clade 2.3.2.1c virus in poultry in Cameroon, 2016-2017. *Avian Pathology*, DOI: 10.1080/03079457.03072018.01492087.	Y	Y	I	U	N	Y	I	Y	High
173.	Yemelong, D. W. S. (2019). *Prévalence de la co-infection fasciolose et tuberculose bovine à l'abattoir de Bangangte [Unpublished doctoral dissertation].* (Doctor of Veterinary Medicine), Institut des Sciences de la Santé, Université des Montagnes, Bangangté, Cameroon.	Y	Y	Y	Y	Y	Y	Y	Y	High
174.	Yongue, C. (2014). *Séroprévalence de la peste des petits ruminants dans le département du Ndé [Unpublished doctoral dissertation].* (Doctor of Veterinary Medicine), Institut Supérieur des Sciences de la Santé, Université des Montagnes, Bangangté, Cameroon.	Y	Y	Y	Y	Y	Y	Y	Y	High
175.	Zangho, K. M. S. (2017). *Analyse épidémique post grippe aviaire dans les localités de Bafoussam et de Yaoundé au Cameroun. [Unpublished doctoral dissertation].* (Doctor of Veterinary Medicine), School of Veterinary Medicine and Sciences, University of Ngaoundere, Cameroon.	Y	Y	Y	Y	Y	Y	Y	Y	High
